# A Heart for Diversity: Simulating Variability in Cardiac Arrhythmia Research

**DOI:** 10.3389/fphys.2018.00958

**Published:** 2018-07-20

**Authors:** Haibo Ni, Stefano Morotti, Eleonora Grandi

**Affiliations:** Department of Pharmacology, University of California, Davis, Davis, CA, United States

**Keywords:** cardiac electrophysiology, physiological variability, computational modeling, arrhythmia mechanisms, big data

## Abstract

In cardiac electrophysiology, there exist many sources of inter- and intra-personal variability. These include variability in conditions and environment, and genotypic and molecular diversity, including differences in expression and behavior of ion channels and transporters, which lead to phenotypic diversity (e.g., variable integrated responses at the cell, tissue, and organ levels). These variabilities play an important role in progression of heart disease and arrhythmia syndromes and outcomes of therapeutic interventions. Yet, the traditional *in silico* framework for investigating cardiac arrhythmias is built upon a parameter/property-averaging approach that typically overlooks the physiological diversity. Inspired by work done in genetics and neuroscience, new modeling frameworks of cardiac electrophysiology have been recently developed that take advantage of modern computational capabilities and approaches, and account for the variance in the biological data they are intended to illuminate. In this review, we outline the recent advances in statistical and computational techniques that take into account physiological variability, and move beyond the traditional cardiac model-building scheme that involves averaging over samples from many individuals in the construction of a highly tuned composite model. We discuss how these advanced methods have harnessed the power of big (simulated) data to study the mechanisms of cardiac arrhythmias, with a special emphasis on atrial fibrillation, and improve the assessment of proarrhythmic risk and drug response. The challenges of using *in silico* approaches with variability are also addressed and future directions are proposed.

## Introduction

Beginning with the seminal paper by Hodgkin and Huxley, 1952, mathematical models of electrophysiology have proven to be valuable tools for better understanding many physiological processes, especially in cardiac arrhythmia research (Noble et al., 2012; Dibb et al., 2015). Fifty-six years after publication of the first cardiac model (Noble, 1962), there is currently a computational model for almost every cell type of the heart, including nodal, atrial, ventricular, and Purkinje cells (Beeler and Reuter, 1977; Difrancesco and Noble, 1985; Luo and Rudy, 1991; Inada et al., 2009; Maltsev and Lakatta, 2009; Sampson et al., 2010; Grandi et al., 2011; O'Hara et al., 2011), for numerous species, and for various levels of complexity across multiple spatial scales (e.g., inclusion of disease-associated remodeling, drug action, contractile, energetics, and signaling modules) (Fink et al., 2011). Most of these models use average data from voltage-clamp experiments of individual ionic membrane currents, and while they have led to many important advances in studies of cardiac electrophysiology and pathology, especially cardiac arrhythmias (Sepulveda et al., 1989; Courtemanche and Winfree, 1991; Panfilov and Holden, 1991; Gray et al., 1995; Krogh-Madsen and Christini, 2012; Roberts et al., 2012; Bueno-Orovio et al., 2014), they typically represent the average behavior of a particular cell type. Because these models ignore evident inter- and intra-personal variability, they can fail to capture the properties of any given individual cell or cells in a given patient (Golowasch et al., 2002; Dokos and Lovell, 2004; Sarkar and Sobie, 2010; Marder, 2011; Zaniboni, 2011; Groenendaal et al., 2015; Pathmanathan et al., 2015). This is in part because incorporating variance that reflects biological data into cardiac models requires significant computational capacity, particularly as compared to what was available when mathematical modeling of electrophysiology first emerged. Given ever-increasing computational capabilities, new modeling approaches have been developed to reproduce and analyze the immense physiological diversity observed in electrophysiology.

In this review, we discuss the importance of accounting for variability when building models of cardiac electrophysiology in both physiological and diseased conditions, and describe new tools being developed to address the limitations of traditional modeling approaches. In particular, we focus on two computational approaches that have emerged as leading methodologies for studying variability in cardiac electrophysiology, which we will refer to as (1) population-based modeling and (2) sample-specific modeling. Both methodologies have provided valuable insights in the fields of neuroscience, cardiology, and pharmacology. Here we review how they have advanced our understanding of the basic mechanisms of cardiac arrhythmias, and particularly atrial fibrillation (AF). As these *in silico* approaches have led to important insights into arrhythmia risks, mechanisms of arrhythmogenesis, and variable response to drugs, they should be considered when determining the regulatory requirements for the proarrhythmia assessment and drug efficacy and safety evaluation of candidate compounds.

## Importance of taking into account variability in cardiac electrophysiology

Sources of variability in cardiac electrophysiology encompass multiple spatial and temporal scales, and include inter-species (Sham et al., 1995; Su et al., 2003), inter-ethnic (Lau et al., 2012; Fender et al., 2014), inter-subject (Taneja et al., 2001; Batchvarov et al., 2002), regional (Feng et al., 1998; Yan et al., 1998), and temporal (Jeyaraj et al., 2012) differences. Variability in experimental electrophysiological data does not only represent natural physiological diversity, but also reflects, in part, differences in the experimental conditions in which electrophysiological measurements are performed (Groenendaal et al., 2015). These conditions can vary from laboratory to laboratory (Niederer et al., 2009; Fink et al., 2011), experiment to experiment, or during the same experiment, e.g., due to deterioration of the experimental preparations over time (Fink et al., 2011). There is also instrument noise (Mirams et al., 2016), artifacts, and use of non-physiological temperatures and solutions (Groenendaal et al., 2015), all of which impact the structure and function of the cellular or tissue components being studied. These sources of variation are difficult to control even for experienced electrophysiologists and are equally as challenging to account for by modelers.

Mathematical cardiomyocyte models have remained useful tools for unraveling physiological and pathophysiological mechanisms, including mechanisms of arrhythmia, and identifying antiarrhythmic strategies without accounting for variability (Sepulveda et al., 1989; Courtemanche and Winfree, 1991; Clancy and Rudy, 1999, 2002; Clancy et al., 2002; Rivolta et al., 2002; Gong et al., 2007; Noble et al., 2007; Tsujimae et al., 2007; Zhang et al., 2007; Zhu and Clancy, 2007; Campbell et al., 2008; Comtois et al., 2008; Kharche et al., 2008; Sale et al., 2008; Ahrens-Nicklas et al., 2009; Butters et al., 2010; Adeniran et al., 2012; Edwards et al., 2014; Grandi and Maleckar, 2016; Morotti et al., 2016; Ni et al., 2017). Although average models have also been successfully applied to the study of sources of variability, such as sexual and hormonal factors (Yang et al., 2010, 2017; Yang and Clancy, 2012), age (Behar and Yaniv, 2017), and circadian regulation (Fotiadis and Forger, 2013), the rationale for developing novel computational approaches that specifically account for electrophysiological variability can be summarized by two main reasons.

### Average data may not accurately represent any specific individual or behavior well

The traditional cardiac model-building scheme involves averaging over samples from multiple experiments from many individuals, both to parameterize the model and validate it, which may not represent any specific measured physiological behavior very well. This “failure of averaging” has been demonstrated in many fields, most recently in neuroscience (Golowasch et al., 2002; Marder, 2011), and was particularly well-documented in 1952, the same year that the seminal Hodgkin and Huxley paper was published, when Lt. Gilbert S. Daniels of the U.S. Air Force published a Technical Note that highlighted the fundamental problem with fitting data to the mean (Daniels, 1952; Rose, 2017). Using data from 4,063 pilots, Lt. Daniels calculated the average of 10 physical dimensions believed to be most relevant for design of the cockpit on a plane, including a pilot's height, chest circumference, and sleeve length. Surprisingly, he found that a total of zero individuals fit within the middle 30% of the range of values for each dimension, and less than 3.5% of pilots would be average sized on any three dimensions. After this finding, the U.S. Air Force completely moved from standardizing all dimensions to an “average pilot,” to making all the dimensions adjustable to each individual pilot, which immediately and drastically improved performance and was soon adopted by all branches of the American military. Modeling of electrophysiology is undergoing a similar evolution, which will likely improve the translational significance of the models.

### Variability has implications on genesis and treatment of arrhythmia

**Figure 1:**
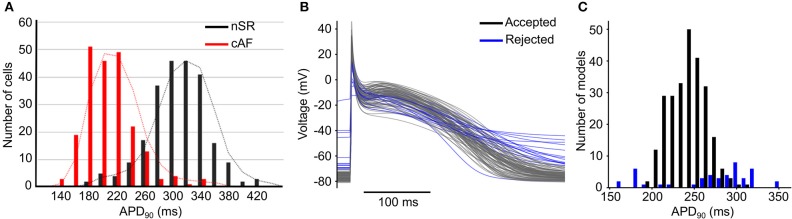
Variability in cardiac electrophysiology.**(A)** Histograms of AP duration at 90% repolarization (APD_90_) for patients in nSR (*black*) and cAF (*red*) show substantial variability. Reproduced from Ravens et al. (2015) with permission. **(B)** Example of APs and **(C)** histogram of APD_90_ produced using models incorporating variability in conductances of ionic currents; some models (*in blue*) are rejected due to non-physiological behaviors.

Variability plays an important role in arrhythmia generation and treatment, as exemplified by AF. The atria are characterized by a high degree of phenotypic variability in physiological properties, with broad and diverging distributions of biomarkers in patients in normal sinus rhythm (nSR) or chronic AF (cAF, Figure 1A) (Ravens et al., 2015), likely due to innate variability of the ionic currents (perhaps due to stochastic gating) that can affect whole cell and/or tissue proarrhythmic behavior (Pueyo et al., 2011; Heijman et al., 2013). This phenotypic variability can be captured by adding variability in the conductance parameters of a mathematical model of the action potential (AP, Figures 1B,C). In some circumstances, physiological variability itself can be the substrate for arrhythmia. For example, increased heterogeneity of refractoriness is important for the maintenance of AF (Moe et al., 1964; Boutjdir et al., 1986; Misier et al., 1992; Sato et al., 1992; Wang et al., 1995, 1996; Gaspo et al., 1997; Liu and Nattel, 1997; Ramirez et al., 2000), and regional differences in atrial ionic currents play a significant role in atrial arrhythmia initiation (Feng et al., 1998; Gaborit et al., 2007; Colman et al., 2013). Consequently, pharmacotherapy that increases dispersion of refractoriness is an adverse side effect of drugs for the treatment of AF (Ramanna et al., 2001; Soylu et al., 2003).

It is well-known that individuals may present largely different responses to same pharmacological interventions. As an example, it has been shown that drugs that block the hERG (human *ether-à-go-go*-related gene) channel are generally responsible for drug-induced long QT syndrome (diLQTS), but in a population this adverse response is highly variable, from minimum changes in the electrocardiogram (ECG) to induction of lethal ventricular arrhythmias (Singh et al., 2000; Kannankeril et al., 2011). Accounting for physiological variability may help better understand why some individuals display adverse side effects, while others do not. Given the different etiologies of many cardiac arrhythmias, such as AF, computational approaches that take into account variability may help us identify subpopulations in which a particular antiarrhythmic therapy will be effective and safe, or toxic. Furthermore, when assessing the efficacy and safety of a drug administration for heart conditions, it is important to take into account physiological and pathological variabilities to make sure that results are quantified and valid at the population level. Such approaches will potentially be more clinically useful in simulating the effects of drugs and aiding the design of safer and more effective therapies (Britton et al., 2013, 2017a; Passini et al., 2016; Yang et al., 2016).

## Approaches and insight on the impact of variability on cardiac electrophysiology

**Figure 2:**
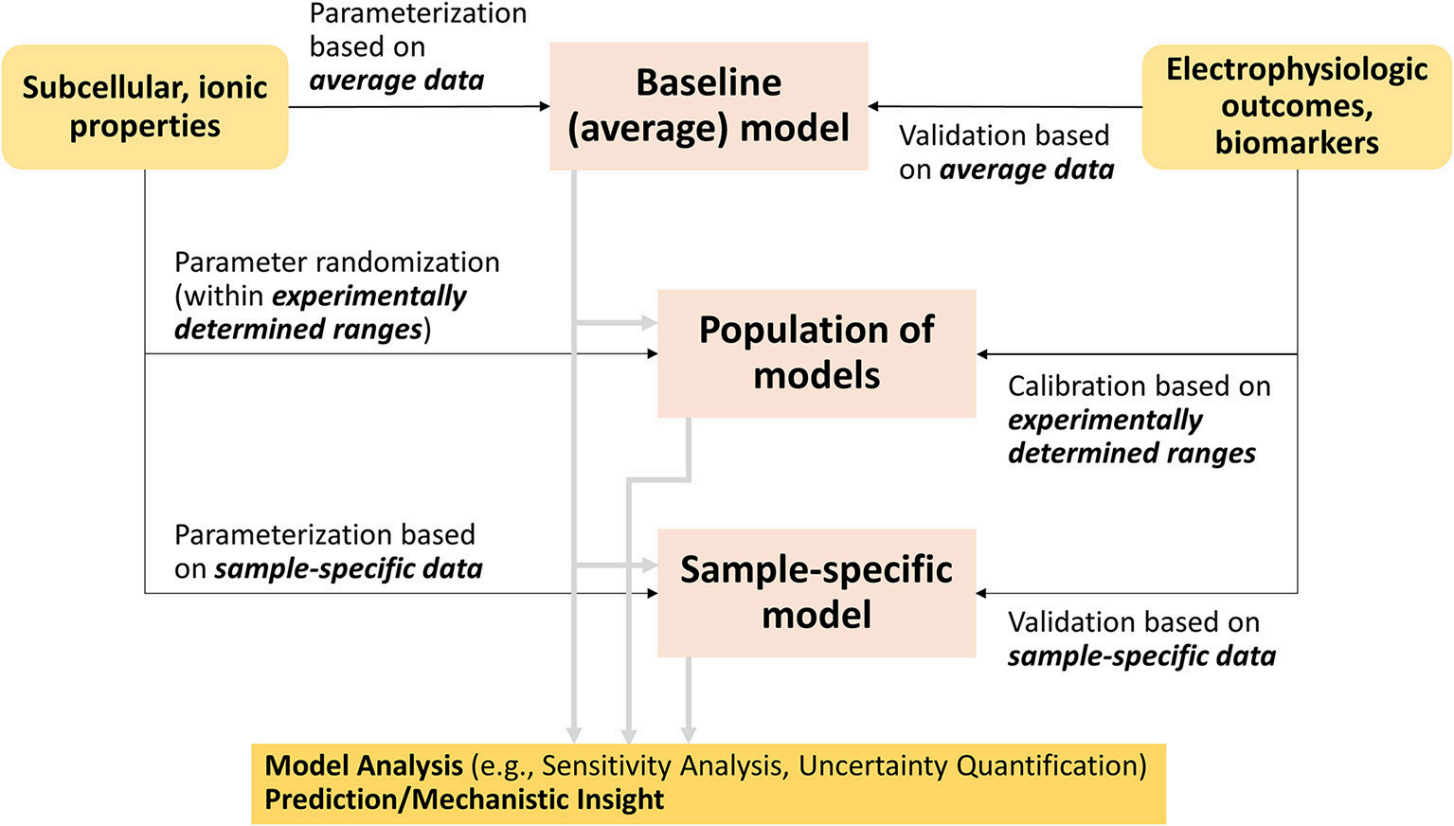
Flowchart connecting traditional cardiac modeling approach to the new methodologies that account for variability.

Although many methods have been developed, two families of approaches have emerged as leading methodologies to account for variability in cardiac electrophysiology: (1) population-based and (2) sample-specific modeling (Figure 2). Both methods generally start with the building or use of a baseline cardiac cell model, which has been parameterized and validated to average data. Population-based approaches generate model variants of the baseline model that fit given experimental distributions of electrophysiological outcomes or biomarkers, while sample-specific modeling approaches re-parameterize the baseline model based on cell- or patient-specific datasets (Figure 2). Because their implementation requires computational power, the advancements in computing capabilities and techniques (Pitt-Francis et al., 2006; Abramson et al., 2010), especially in parallel computing (Wang et al., 2011), have helped these methods gain traction in the last decade.

### Population-based modeling

Population-based modeling approaches have been developed and employed to obtain results at the population level, which led to many novel insights into physiological and pathophysiological variabilities, and variable responses to drug administration. We refer the readers to a recent review from the Rodriguez group (Muszkiewicz et al., 2016), where this methodology is described in detail. Here, we briefly describe the general approach of population-based modeling, and summarize how it has contributed to advancing the field as exemplified by some important studies.

#### Creating populations of AP models

Populations of models are generally created by modifying sets of parameters in a baseline model (Figure 2). This process involves determination of the parameters to be varied, over what range, and a sampling method to select the parameter values. Frequently, maximal conductances or maximum transport rates of ion channels, pumps and transporters in AP models are selected to vary. The parameter space over which these model parameters vary can be chosen either to reflect the experimental range, when available, or theoretical upper and lower bounds. Then, populations of parameter sets are created by sampling the parameters within the predefined parameter spaces. Typically, four types of sampling methods have been applied to obtain the populations of parameter sets: uniform-interval sampling (Romero et al., 2009, 2011; Corrias et al., 2011), log-normal sampling (Sobie, 2009; Sadrieh et al., 2013; Ellinwood et al., 2017a; Morotti and Grandi, 2017), Latin hypercube sampling (LHS) (Britton et al., 2013) and its variants such as orthogonal sampling (Burrage et al., 2015; Donovan et al., 2018), and sequential Monte Carlo sampling (Muszkiewicz et al., 2017).

After generating hundreds or thousands of model variants, calibration can be performed to exclude models that display non-physiological behaviors (Figure 2). This can be done, for example, by removing models showing repolarization failure (Sobie, 2009), or exhibiting AP duration (APD) more than three standard deviations from the population mean (Devenyi and Sobie, 2016). Population of models are also calibrated to measured data from patient samples, whereby model variants are selected based on simulated electrophysiological properties, such as APD, upstroke velocity, resting membrane potential and/or Ca^2+^ transient (CaT) (Britton et al., 2013, 2017a; Sanchez et al., 2014; Passini et al., 2016; Rees et al., 2018). Other studies use additional information such as ionic current data (Muszkiewicz et al., 2017), or ECG data (Mann et al., 2016). This calibration step is meant to ensure that (1) variants displaying non-physiological properties are discarded before analysis, and (2) the simulated electrophysiological properties of models in a given population are in the same range as experimental data, or, more recently, correspond to the same distribution of observed experimental biomarkers (Lawson et al., 2018), thus possibly making inferences from *in silico* experiments more physiologically relevant.

#### Analyzing populations of AP models

Once a population of cardiac AP models is generated, and electrophysiological simulations have been performed corresponding to the scientific question at hand, mechanistic insights can be obtained using various analysis techniques. These analyses have contributed to our understanding of the relative role of the underlying parameters in modulating the physiological properties of interest (i.e., sensitivity analysis), or revealing association of certain parameter ranges or properties with specific physiological behaviors (e.g., repolarization abnormalities, ectopic activity, drug response). Many relevant examples have recently been reviewed (Muszkiewicz et al., 2016). Here we highlight new recent developments and discuss details of parameter sensitivity analysis.

##### Performing parameter sensitivity analysis

A common systematic analysis of populations of models is sensitivity analysis. It assesses how model outputs, which typically represent whole cell behavior (e.g., APD), are sensitive to changes in model parameters, (e.g., conductances and maximum transport rates). Because many parameters are often varied to generate the populations of models, multivariable linear regression (Hair et al., 2010; Draper and Smith, 2014) has emerged as a frequently utilized tool to perform sensitivity analysis in cardiac electrophysiology. Moreover, as the number of independent parameters varied is used to predict a smaller set of dependent variables, sensitivity analysis is typically performed using partial least squares regression (Geladi and Kowalski, 1986; Sobie, 2009), as compared to standard multivariable regression. The result of linear regression is a set of coefficients (forming a “regression model”) describing how perturbing a particular parameter influences an output of interest. This method has been successfully utilized in other fields such as molecular biology (Janes et al., 2005) and neuroscience (Weaver and Wearne, 2008), and was first used in cardiac electrophysiology by Sobie (2009), who applied it to study sensitivities of properties such as APD and pacing rate threshold for inducing AP alternans. Since the regression model represents a linear approximation of a highly non-linear system, it is important to always check the goodness of fit. Several papers by the Sobie's group have indeed shown that the linear approximation actually provides a very good fit of the AP biomarkers, which was not trivially predictable (Sarkar et al., 2012).

The approach of varying multiple ionic conductances at once in a systematic fashion (as opposed to one at a time) and employing sensitivity analysis using multivariable regression has led to many important insights in cardiac electrophysiology (Sarkar and Sobie, 2011; Mann et al., 2012; Heijman et al., 2013; Walmsley et al., 2013), some of which have been confirmed experimentally (Lee et al., 2013; Devenyi and Sobie, 2016; Devenyi et al., 2017). For example, it has been used to study how different diseased conditions affect the sensitivities of given electrophysiological properties (Sadrieh et al., 2013; Ellinwood et al., 2017a; Vagos et al., 2017; Koivumaki et al., 2018), mechanisms of physiological phenomena (Lee et al., 2013), and for constraining free parameters (Sarkar and Sobie, 2010). Through sensitivity analysis, Cummins et al. identified multiple potential ionic targets mediating forward rate dependence (FRD) of AP, and demonstrated that modulation of the inward rectifier K^+^ current (I_K1_) or the Na^+^/K^+^ pump current was more likely to produce FRD (Cummins et al., 2014). Devenyi and Sobie performed sensitivity analysis of rat ventricular myocyte models, and quantitatively compared the modulatory role of transient outward K^+^ current (I_to_) and sarco/endoplasmic reticulum Ca^2+^-ATPase (SERCA) in CaT amplitude. They found that in rat epicardial cells I_to_ plays a more important role than SERCA in regulating CaT amplitude, and this was analogous to human atrial cells, where both I_to_ and ultra-rapid delayed-rectifier K^+^ current (I_Kur_) had greater impacts on CaT amplitude than did SERCA (Devenyi and Sobie, 2016). These studies highlight how sensitivity analysis can be applied to compare and contrast roles of different ionic processes and Ca^2+^ handling in regulating physiological properties and behaviors between cell types and species. Sensitivity analysis has also been used to compare the dependence of AP biomarkers on ionic processes in healthy and diseased conditions. For example, Lee et al. compared the impact of ionic processes on APD in control and AF-remodeled cells and found that the Na^+^/Ca^2+^ exchanger (NCX) current has little influence on APD in control cells but more markedly impacts AF cells; the analysis also revealed that I_K1_ upregulation plays a dominant role in APD shortening in AF, and that the L-type Ca^2+^ current (I_CaL_) significantly contributes to rate-dependent APD changes in both control and AF myocytes (Lee Y. S. et al., 2016). Most recently, Gong and Sobie described a novel use of regression models, cross-cell regression, to predict adult myocyte drug responses from induced pluripotent stem-cell-derived cardiomyocytes (iPSC-CMs) behaviors (Gong and Sobie, 2018).

Multivariable linear regression is used if the physiological output of interest is continuous, but, for the study of arrhythmia mechanisms, another particularly useful and efficient regression technique is logistic regression, which is used when the outcome of interest is Boolean (i.e., yes/no, true/false). Applying logistic regression in studies of physiology is well-described by Lee et al. who examined Ca^2+^ spark triggering (an all-or-none event), and demonstrated the accuracy of logistic regression using receiver operator characteristic curves (Lee et al., 2013). This method has since been used to study the probability that a certain arrhythmic event such as early afterdepolarizations (EADs) will occur and suggest underlying factors (Morotti and Grandi, 2017).

**Figure 3:**
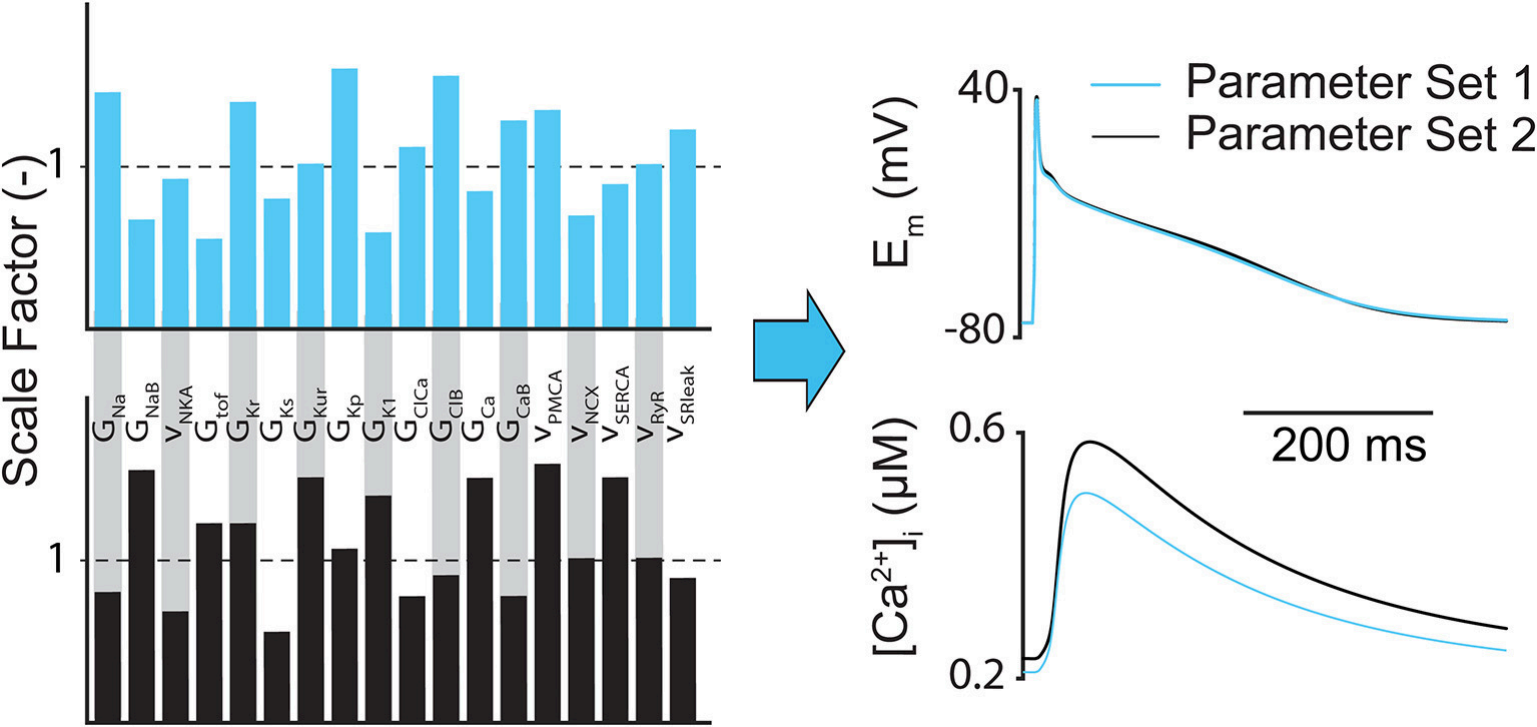
Different subcellular parameter combinations can lead to same AP shape. Example of how different model parameter combinations (e.g., ion channel conductances and maximum transport rates) can produce nearly identical atrial AP morphologies, but notably different CaTs.

The main limitation of regression (both linear and logistic) analysis is that it only highlights how inputs are correlated to outputs, so the conclusions drawn from the analysis can be misleading if only a few outputs are considered. For example, it has been shown that completely different parameter combinations could produce essentially identical AP shapes but substantially different CaT amplitudes (Figure 3) (Sarkar and Sobie, 2010). However, sensitivity analysis can still help determine whether the relationships between inputs and outputs in computational models match experimental findings and assumptions, and whether there are particularly influential parameters that can be exploited therapeutically or targeted to better understand a given physiological phenomena (e.g., arrhythmia mechanism).

##### Comparing subpopulations of models

Comparing subgroups in a population of models (often using statistical difference tests of parameters of interest) can help identify underlying determinants of different phenotypes, behaviors, and pathological conditions (Sanchez et al., 2014; Zhou et al., 2016; Britton et al., 2017b; Muszkiewicz et al., 2017; Vagos et al., 2017; Lawson et al., 2018). For example, through characterizing ionic parameters of models that are prone to repolarization abnormalities, Britton et al. found that the electrogenic Na^+^/K^+^ pump is a key determinant of susceptibility to repolarization abnormalities in human ventricular cardiomyocytes by applying arrhythmia-provoking conditions to a population of experimentally-calibrated cardiac cells (Britton et al., 2017b). A population-based approach has also been used to tease out the ionic mechanisms underlying variability in iPSC-CMs (Paci et al., 2017). By calibrating generated subpopulations of human atrial myocyte models to ranges of experimental data from a large number of patients with nSR or cAF, Sanchez et al. characterized potential ionic determinants of inter-subject variability in AP biomarkers, and identified similar changes in I_K1_, I_Kur_, and I_to_ in cAF vs. nSR subpopulations that were consistent with experimentally reported AF-induced remodeling effects (Sanchez et al., 2014). In a more recent study, instead of calibrating population of models to the range of experimental dataset, Lawson et al. proposed a novel method to calibrate these models to the distributions of multiple experimentally measured biomarkers (Lawson et al., 2018), which led to an improved characterization of ionic differences between nSR and cAF. These studies focused on AP biomarkers at a fixed pacing rate. In a different study, Vagos et al. expanded the use of population of models to compare the steady state and dynamic restitution behaviors of AP in nSR and cAF populations (Vagos et al., 2017). By combining population-based modeling and experiments, Muszkiewicz et al. characterized variability in AP and ionic densities and their impact on CaT in atrial cells from right atrial appendage of patients exhibiting nSR (Muszkiewicz et al., 2017). In addition to calibrating model outputs to measured AP biomarkers, they also extended the experimental calibration of population of human atrial models to model parameter (inputs) by using experimental data of major ionic currents.

##### Quantifying drug modulatory effects, understanding variability in drug response, and identifying phenotype-specific therapy

By using a population of models that incorporate variabilities, drug modulatory effects on electrophysiological properties can be interpreted at a whole population level, which also contributes to limiting potential model-dependent results. For example, Yang et al. used a population-based approach to simulate effects of late Na^+^ current (I_NaL_) and hERG block and found that the selective I_NaL_ blocker GS-458967 could suppress proarrhythmic markers after hERG block in ventricular myocytes (Yang et al., 2016). Population-based modeling has also allowed for more rigorous quantitative comparison of modulatory effects between multiple drugs. A recent study by Britton et al. calibrated populations of ventricular models to specific individuals using data from human trabeculae (Britton et al., 2017a). They then assessed the effects of four different (selective and non-selective) blockers of the rapid delayed-rectifier K^+^ current (I_Kr_), dofetilide, sotalol, quinidine, and verapamil, to quantitatively compare changes in AP biomarkers, and demonstrated good agreement with experiments for the selective I_Kr_ blockers (dofetilide and sotalol) but not for the non-selective I_Kr_ inhibitors (quinidine and verapamil). Paci et al. utilized populations of *in silico* iPSC-CMs to evaluate antiarrhythmic effects of mexiletine and ranolazine to treat iPSC-CM long QT syndrome type 3 (LQT3) mutants and demonstrated that mexilitine stops spontaneous APs in more LQT3 models than ranolazine due to its stronger effects on the fast Na^+^ current (I_Na_) (Paci et al., 2017). In contrast to the traditional modeling approach using a single model, the population-based modeling can gain insights into the physiologically relevant variability of predictions made *in silico*, as demonstrated in these studies.

By taking a step further, simulations using populations of models incorporating variabilities can also help recognize the contributing factors underlying the variability observed in response to drugs. One relevant example is the variable outcomes of hERG inhibition, which is frequently linked with diLQTS. Population-based modeling has offered insights into the mechanisms underlying the fact that individuals will not exhibit the same degree of QT interval prolongation after hERG block (Singh et al., 2000; Kannankeril et al., 2011; Weeke et al., 2014). Employing a population of models of ventricular myocytes, Sobie and Sarkar attributed the variable outcomes to the different ionic properties of the cells (Sarkar and Sobie, 2011). In another interesting application, Passini et al. implemented an *in silico* drug trial using experimentally-calibrated populations of AP models to investigate the risk of drug-induced arrhythmias, and to identify specific subpopulations at higher risk for proarrhythmic cardiotoxicity (Passini et al., 2017). Their methodology not only demonstrated higher accuracy than animal models in predicting arrhythmia risk, but also provided mechanistic insight into the underlying ionic contributors to repolarization/depolarization abnormalities.

Understanding the bases of variability in electrophysiological behavior and arrhythmia proclivity may also allow developing specific antiarrhythmic therapies for different disease phenotypes. For example, Liberos et al. compared AF models that had self-sustained vs. self-terminating reentries (Liberos et al., 2016). They found that AF maintenance was correlated with high I_CaL_ and I_Na_, and that I_CaL_ block could be an effective treatment depending on the basal availability of Na^+^ and Ca^2+^ ion channel conductivities (I_Na_ depression increased efficacy). Mayourian et al. employed a comprehensive integrated approach to study the mechanisms of cardiac contractility and arrhythmogenicity using experimentally-calibrated human mesenchymal stem cells (hMSCs) (Mayourian et al., 2017). In simulations testing proarrhythmic effects, they found that hMSCs paracrine signaling protected such adverse effects of heterocellular coupling at various levels of engraftment. This work highlights that antiarrhythmic strategies can move beyond simply considering repolarization abnormalities.

### Sample-specific modeling

Instead of taking a baseline cardiac model and introducing variability by randomly varying the ionic conductances, optimization and statistical techniques can also be used to tailor the baseline model to describe a specific experimental sample. Depending on the characteristics of the dataset at hand, sample-specific models can be representative of either individual myocytes or a particular group of cells. The former approach, cell-specific modeling, can be helpful when integrating mathematical modeling into an experimental setup. For example, Ravagli et al. characterized the role of the “funny” current I_f_ in sinoatrial myocytes using the dynamic clamp technique by adapting the extent of injected I_f_ in a cell-specific fashion, i.e., based on the basal firing rate measured in each individual cell (Ravagli et al., 2016). Despite the use of average data, sample-specific models built from a group of cells (e.g., a cell line developed in a certain laboratory, myocytes isolated from disease models, iPSC-CMs derived from a single patient) can allow for specific characterization of conditions that are far from the average, or even of personalized physiology (Barichello et al., 2018). For example, monophasic AP data recorded in AF patients undergoing ablation procedures have been used to construct atrial cell models that capture patient-specific electrophysiological properties (Lombardo et al., 2016). This approach has the promise of making patient-specific predictions given interventions such as arrhythmia-provoking protocols or drug application. Here we summarize methodologies for building and improving sample-specific cell models. For more detail, we refer the readers to a previous review on the topic (Krogh-Madsen et al., 2016).

#### Fitting sample-specific models

Sample-specific models can be constructed by fitting the parameters of a baseline model so that the model outputs match the corresponding physiological behaviors seen in a single patient or myocyte (Figure 2). Cardiac electrophysiology models can be optimized using many different fitness functions (Druckmann et al., 2007; Tomaiuolo et al., 2012), such as global search heuristics (Vanier and Bower, 1999; Dokos and Lovell, 2004; Bueno-Orovio et al., 2008; Guo et al., 2010). Recently, many sample-specific models are generated using the genetic algorithm (GA), which traces its beginnings to evolutionary biology (Fraser and Burnell, 1970; Crosby, 1982), but is still being applied in new ways today (Chen and Guan, 2004; Hussein and El-Ghazaly, 2004; Leung et al., 2004; Vieira et al., 2004). Its use for optimization of ionic models is relatively new in both neuroscience (Achard and De Schutter, 2006; Gurkiewicz and Korngreen, 2007; Hobbs and Hooper, 2008; Ben-Shalom et al., 2012) and cardiac electrophysiology (Syed et al., 2005; Bot et al., 2012; Kaur et al., 2014; Groenendaal et al., 2015). Syed et al. demonstrated its feasibility for atrial cell models when they proved they could fit two different cell models (Courtemanche et al., 1998; Nygren et al., 1998) to any given atrial AP (Syed et al., 2005). Essentially, the GA optimization procedure is initialized in the same way as for the population-based approach (varying maximal conductance and/or transport rates), and then it iteratively evolves toward better solutions in generations, while the underlying parameters can be varied, swapped, or discarded. Sensitivity analysis can be used in conjunction with generating sample-specific models as it can inform the design of the error function (i.e., weights) by revealing the conductances that more significantly impact the electrophysiological outputs used for fitting. For example, Krogh-Madsen et al. recently combined sensitivity analysis and global optimization (using a GA) of a ventricular myocyte model to clinical long QT data and intracellular Ca^2+^ and Na^+^ concentrations, to better constrain the model parameters (Krogh-Madsen et al., 2017). They found that this improved prediction of drug-induced *torsades de pointes* (TdP), especially in eliminating false-positive outcomes generated by the baseline model parameters.

#### Improving fidelity of sample-specific models

The final solution of an optimization procedure using some fitness function may not match experimental data well if only fitting to a single electrophysiological output such as a single AP, because multiple parameter combinations can potentially produce the same AP (Figure 3) (Syed et al., 2005; Druckmann et al., 2007; Sarkar and Sobie, 2010; Guo et al., 2013; Kaur et al., 2014; Groenendaal et al., 2015). In this case, although fitness function itself can be improved, for example, by increasing the population size or diversity for GAs can improve the fit of a sample-specific model, it may not necessarily guarantee that the final solution relates to the global minimum. To address this issue, many methods have been developed using (1) additional electrophysiological properties for fitting and/or (2) more complex electrophysiological protocols to improve model fidelity. It has been shown that model faithfulness can be improved by adding membrane resistance as an objective (Kaur et al., 2014), by optimizing to Ca^2+^ handling (Dokos and Lovell, 2004; Sarkar and Sobie, 2010; Rees et al., 2018), or by accounting for experimental data generated from multiple pacing frequencies (Syed et al., 2005; Lombardo et al., 2016) or irregular pacing protocols (Guo et al., 2013; Groenendaal et al., 2015).

**Figure 4:**
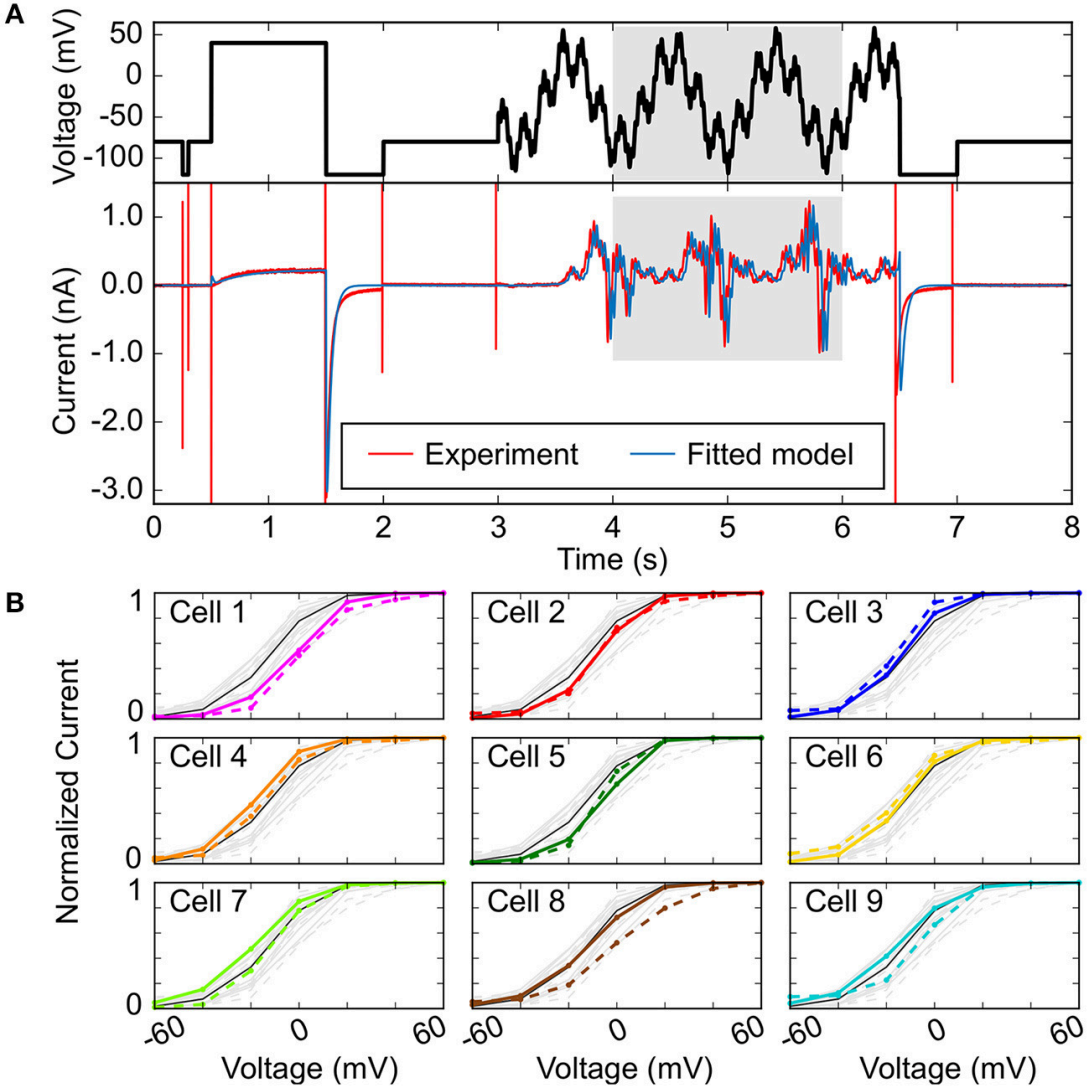
Improving fit of sample-specific models**. (A)** Experimental and simulated I_Kr_ time courses (bottom) evoked in response to an efficient, information-rich sum-of-sinusoid voltage protocol (top) that allows rapid characterization of I_Kr_ behavior. **(B)** Steady-state peak I_Kr_-voltage curves comparing cell-specific model predictions (bold, colored) to cell-specific experimental recordings (dashed, colored). The black lines in each plot are from the model calibrated to averaged sinusoidal data from all the cells (light gray). Reproduced from Beattie et al. (2018) with permission.

In addition to using multiple electrophysiological properties to improve the fit of sample-specific models, more intricate voltage-clamp protocols that capture complex and rich electrophysiological dynamics have been employed, as first demonstrated to improve the fit of Markov models of ionic currents with many parameters (Dokos and Lovell, 2004; Zhou et al., 2009; Beattie et al., 2018). These can be implemented over a short time frame and may be used to emphasize certain currents over others. In the absence of pharmacological isolation, Groenendaal et al. used only a 6-s protocol that effectively isolated I_K1_, I_CaL_, and slow delayed-rectifier K^+^ current (I_Ks_) given their disproportionately large contribution at voltage steps of −120, +20, +40, and −30 mV, respectively (Groenendaal et al., 2015). They found that using this protocol alone cannot isolate all ionic currents, and when used in combination with a stochastic pacing protocol there was a considerable improvement in parameter estimation. Developing short but information-rich protocols is useful especially when trying to improve the results of an optimization procedure for cell-specific modeling, because longer protocols take longer to implement experimentally and thus are difficult to perform in a single cell. In a recent study, Beattie et al. proposed an innovative experimental and mathematical modeling method that allows to concisely measure the dominant processes involved in hERG channel gating by applying a short (8-s long) “sum of sinusoids” voltage-clamp protocol (Beattie et al., 2018). The sinusoidal waves were able to provoke a wider range of non-equilibrium behavior than traditional square voltage steps, thus allowing rich and complete characterization of hERG channel kinetics in the same cell and efficient model fitting (Figure 4A).

The final step in improving fidelity of sample-specific models is to directly experimentally test the predictions of the model given new protocols (Groenendaal et al., 2015; Devenyi et al., 2017; Beattie et al., 2018). Figure 4B reports an example of such validation experiments, where predictions obtained with cell-specific I_Kr_ models (identified applying the sinusoidal protocol in Figure 4A in 9 cells) are compared to the I_Kr_-voltage relationships experimentally determined in each cell (Beattie et al., 2018). The order of the panels in Figure 4B is based on an index of recording stability (lowest to highest difference in leak resistance between the vehicle and dofetilide recordings) that is associated to “data quality”. Cell-specific predictions are excellent for cells 1–5 (higher data quality), but less accurate for cells 6–9 (lower data quality). The analysis also shows that cell-specific models provide better predictions than the average model for the cells with the highest data quality (cells 1–5). Experimental validation is an important last step in improving cell-specific models, as generating cell-specific models is potentially more susceptible to observational error. Devenyi et al. used a GA to re-parameterize the Livshitz-Rudy model of the guinea pig ventricular cardiomyocyte (Livshitz and Rudy, 2009), and predicted an increase in I_Kr_ and a drastic decrease in I_Ks_ given a dynamic clamp protocol as compared to the original model, and this was validated experimentally (Devenyi et al., 2017). Their adjusted model predicted that I_Ks_ can stabilize the AP and EADs better as compared to I_Kr_—which improved the ability to assess arrhythmia risk, given the baseline model did not produce physiological behaviors that were quantitatively similar to their experiments.

#### Models of patient-specific anatomy

While a detailed discussion of patient-specific anatomical models is beyond the scope of our review, recent studies have begun investigating how inter-patient differences in myocardial structure affects atrial arrhythmia, as reviewed by Barichello et al. (2018). For example, Zhao et al. developed a 3D human heart-specific atrial computer model integrating 3D high resolution structural and functional mapping data to test the impact of wall thickness, fibrosis, and myofiber orientation on AF induction, maintenance, and ablation strategies (Zhao et al., 2017). Deng et al. demonstrated that reentrant driver localization dynamics are influenced by inter-patient variability in the spatial distribution of atrial fibrosis, APD, and conduction velocity (Deng et al., 2017). This suggests that incorporating patient-specific electrophysiological models in patient-specific geometries might enhance their predictive value. We discuss the computational challenges associated to this task in the section entitled “Arrhythmia Research Requires Understanding Variability at Larger Spatial Scales”. Furthermore, obtaining patient-specific electrophysiological data might constitute another logistical roadblock.

Overall, methods that incorporate variability are particularly useful for (1) analyzing variability in cardiac electrophysiology, (2) assessing proarrhythmic risk, (3) determining the underlying factors contributing to variable drug response, and (4) identifying phenotype-specific (and in the future patient-specific) antiarrhythmic targets. Table 1 summarizes applications of these approaches and new insights provided by the studies (shaded areas indicate atrial studies).

**Table 1:** Applications and main findings of computational methods incorporating cardiac electrophysiological variability (**shaded areas indicate atrial studies).

**Author, Year**	**Baseline Model**	**Model Generation**	**Approach**	**Insight**
**ANALYZING VARIABILITY**			
**Sobie**, **2009**	MVMMs: Luo and Rudy, 1991; Fox et al., 2002; Kurata et al., 2005	Sampled from LND	MLR	New method to rapidly identify ionic mechanisms shaping AP properties, CaT, and alternans
**Sanchez et al.**, **2014**	HAMMs: Courtemanche et al., 1998; Maleckar et al., 2009; Grandi et al., 2011	Sampled over a ±100% variation range around their baseline values as described by Marino et al. (2008); calibrated to AP recordings in atrial trabecula	Statistical difference tests	Ionic determinants of variability in human AP in nSR vs. cAF
**Lee Y. S. et al.**, **2016**	HAMM: Courtemanche et al., 1998	Sampled from LND	MLR	Comparison of parameter sensitivity between nSR and AF condition. Ionic contributions to rate-dependence and spiral wave dynamics in AF
**Devenyi and Sobie**, **2016**	HAMM: Grandi et al., 2011; HVMM: Grandi et al., 2010; RVMM: Pandit et al., 2001	Sampled from LND	MLR	In human atrial myocytes, both I_to_ and I_Kur_ had greater impacts on CaT amplitude than did SERCA. This was similar in rat left ventricular epicardial cells, where I_to_ played a more important role than SERCA
**Vagos et al.**, **2017**	HAMM: Skibsbye et al., 2016	Sampled from Gaussian distribution	MLR	Ionic determinants of unstable behaviors in nSR vs. cAF
**Ellinwood et al.**, **2017b**	HAMM: Grandi et al., 2011	Sampled from LND	MLR	I_Kur_ impacts APD and effective refractory period more in cAF (even though it is downregulated) vs. nSR
**Muszkiewicz et al.**, **2017**	HAMMs: Courtemanche et al., 1998; Maleckar et al., 2009; Grandi et al., 2011	Sampled using LHS and sequential MC; calibrated to experimental recordings	PCCs, statistical difference tests	Ionic determinants of electrophysiological and CaT properties
**Lawson et al.**, **2018**	HAMM: Courtemanche et al., 1998	Sampled over a ±100% variation range around their baseline values; sequential MC; model calibrated based on distributions of biomarkers estimated from multivariate kernel density estimation	Statistical difference tests	Accurate identification of inherent variability within the experimental population and improved characterization of ionic differences between nSR and cAF
**ASSESSING ARRHYTHMIA RISK**			
**Walmsley et al.**, **2013**	HVMM: O'Hara et al., 2011	MC sampling from a uniform distribution (±30%); calibrated to mRNA expression data in failing and non-failing hearts	MLR	Combination of low SERCA activity and high I_CaL_ conductance impacted the formation of alternans the most in the non-failing heart population, but low hERG conductance was the main contributor to alternans in the failing heart population
**Zhou et al.**, **2016**	HVMM: O'Hara et al., 2011	Sampled using LHS; calibrated to *in vivo* recordings	PCCs, statistical difference tests	I_CaL_ and NCX current determine the cell-to-cell differences in repolarization alternans through intracellular and sarcoplasmic Ca^2+^ regulation
**Britton et al.**, **2017b**	HVMM: O'Hara et al., 2011	Sampled using LHS; calibrated to data in human ventricular trabeculae	Logistic regression, PCCs, statistical difference tests	Na^+^/K^+^ pump is a key determinant of repolarization abnormality susceptibility
**Morotti and Grandi**, **2017**	HAMM: Grandi et al., 2011	Sampled from LND	Logistic regression	EADs are particularly sensitive to conductances of I_Na_, acetylcholine-sensitive and ultra-rapid K^+^ channels, and NCX transport rate
**Devenyi et al.**, **2017**	Guinea pig left ventricular myocyte model Livshitz and Rudy, 2009	Fit using GA; sampled from LND	Dynamic clamp data for fitting, MLR	I_Ks_ is more capable to stabilize AP and EADs as compared to I_Kr_
**IDENTIFYING ANTIARRHYTHMIC TARGETS**			
**Cummins et al.**, **2014**	MVMMs: Luo and Rudy, 1991; Fox et al., 2002; Hund and Rudy, 2004; Ten Tusscher et al., 2004; Ten Tusscher and Panfilov, 2006; Livshitz and Rudy, 2009; O'Hara et al., 2011	Sampled from LND	MLR	I_K1_ and Na^+^/K^+^ pump currents favor forward rate dependence
**Liberos et al.**, **2016**	HAMM: Skibsbye et al., 2016	Sampled using LHS; calibrated to AP recordings in atrial trabeculae in patients with AF	PCCs, statistical difference tests	AF maintenance was correlated to high I_CaL_ and I_Na_, and I_CaL_ block could be an effective treatment depending on the basal availability of Na^+^ and Ca^2+^ channel conductivities
**Passini et al.**, **2016**	HVMM: O'Hara et al., 2011	Sampled using LHS; calibrated to non-diseased and HCM myocytes AP recordings	Analysis of repolarization properties	I_CaL_ re-activation is the key mechanism for repolarization abnormalities in HCM myocytes, and combined NCX, I_NaL_ and I_CaL_ block is effective to partially reverse the HCM phenotype
**Yang et al.**, **2016**	Rabbit ventricular myocyte model Soltis and Saucerman, 2010	Randomly selected within ±10% of nominal value (uniform distribution)	Analysis of TRIaD pro-arrhythmic markers	GS-458967 suppressed proarrhythmic markers following hERG block
**ASSESSING THE VARIABLE RESPONSE TO DRUGS**			
**Sarkar and Sobie**, **2011**	MVMMs: Fox et al., 2002; Hund and Rudy, 2004; Ten Tusscher et al., 2004; Kurata et al., 2005; Grandi et al., 2010	Sampled from LND	MLR	Individuals do not exhibit the same degree of QT interval prolongation due to different ionic ensembles
**Britton et al.**, **2013**	Adapted rabbit Purkinje cell model Corrias et al., 2011	Sampled using LHS; calibrated to experimental data	PCCs	Quantitatively predicted the arrhythmia risk of four concentrations of the K^+^ channel blocker dofetilide; baseline I_Kr_ conductance is the primary determinant of APD prolongation caused by dofetilide
**Lancaster and Sobie**, **2016**	HVMMs: Ten Tusscher et al., 2004; Grandi et al., 2010; O'Hara et al., 2011	Sampled from LND; calibrated to experimental data	PCA, ROC curves, TdP risk scores	TdP risk assessment could be improved by quantifying the impact of multiple cardiac ion channels (even those not typically considered to affect risk)
**Britton et al.**, **2017a**	HVMM: Adapted O'Hara et al., 2011	Sampled using LHS, calibrated to heart-specific *ex vivo* measurements	Coefficients of variation	Good agreement with experiments for selective I_Kr_ blockers, but notable differences for the non-selective I_Kr_ inhibitors
**Passini et al.**, **2017**	HVMM: O'Hara et al., 2011	Sampled using LHS; calibrated to experimental data	TdP scoring system	*In silico* drug trial demonstrated higher accuracy than animal models in predicting arrhythmia risk (89%); underlying ionic contributions to repolarization/depolarization abnormalities
**Krogh-Madsen et al.**, **2017**	HVMM: O'Hara et al., 2011	Fit using GA; optimized to clinical data	TdP risk prediction	TdP risk assessment could be improved by using global optimization methods and multi-variable objectives
**Gong and Sobie**, **2018**	HVMM O'Hara et al., 2011 and human iPSC-CM models Paci et al., 2017	Sampled from LND	Cross-cell MLR	Cross-cell regression predicted adult ventricular myocyte drug responses from the behaviors of an iPSC-CM *in silico* population

*HAMM, human atrial myocyte model; HCM, hypertrophic cardiomyopathy; HVMM, human ventricular myocyte model; LHS, Latin hypercube sampling; LND, log-normal distribution; MLR, multivariable linear regression; MVMM, mammalian ventricular myocyte model; RVMM, rat ventricular myocyte model; PCA, principal component analysis; PCC, partial correlation coefficient; ROC, receiver operator characteristic; MC, Monte Carlo; TRIaD, Triangulation, Reverse use dependence, beat-to-beat Instability of action potential duration, and temporal and spatial action potential duration Dispersion*.

## Challenges and future directions

We reviewed the most common methods used to account for variability in cardiac electrophysiology, which largely fall into the two categories of (1) population-based modeling and (2) sample-specific modeling. These methods complement each other well, as population-based methods can help characterize behavior in a particular patient group (healthy, diseased, stressed, etc.), and sample-specific modeling shows significant promise to develop personalized medical approaches for individual patients. Both methods have led to many important insights into the mechanisms of arrhythmogenesis and antiarrhythmic strategies. However, there are several important limitations to consider, which suggest potential future developments in modeling of cardiac electrophysiology.

### Analysis of electrophysiology from populations of models may require different statistical methods

As opposed to the traditional approach of producing a single value from a single baseline model, models that incorporate variability have allowed statistical methods to be applied that can either ask new scientific questions or quantify the impact of variability on electrophysiological outputs, as performed in experimental studies. While the statistical analysis methods used in experimental studies can be directly applied in the *in silico* population-based studies, differences in the nature of experimental and simulation studies may need to be considered. For example, some population-based techniques generate model population sizes (often sample sizes in the 1000s) that are much greater than could be achieved by experiments (often sample sizes of 3–12) or the traditional cardiac modeling approach alone. Therefore, given similar effects, results produced in the population-based simulations have greater statistical power to detect differences. Furthermore, because even very small effects can reach statistical significance with large samples, physiological significance should be assessed (White et al., 2014). Additionally, when evaluating drug effects on electrophysiology, in simulations of the same virtual cell (a single model out of the population-models) can be used to perform both control and with-drug studies, allowing for paired comparisons, which is often not practical in experimental studies. The methodologies for analyzing and interpreting the “big data” produced by the population of models should be carefully considered and standards should be established going forward.

### Variability does not fully account for uncertainty

Physiologic variability should be thought of in the context of the broader umbrella of uncertainty, which is the confidence (or precision) with which a quantity, such as an electrophysiological output, can be given a value (Mirams et al., 2016). While here we reviewed how cardiac electrophysiology models have begun to account for physiological and experimental variability, uncertainty analysis should determine whether the baseline model itself is a valid representation of its physical system. The extent and rigor of validation during model development affects uncertainty, whereby the broader the set of constraints, e.g., the model recapitulates both voltage and Ca^2+^ responses, their pacing rate-dependence, short- and long-term behavior, the lesser the uncertainty in the obtained parameters. Uncertainty analysis should also verify that the experiments used to construct the model are appropriate. For example, in experiments, voltage-clamp protocols used to characterize ionic currents are often done using non-selective pharmacological block which may have unidentified effects, over a range narrower than the entire physiological range, or on larger cells that are easier to patch-clamp with intrinsically greater than normal maximal conductances (Courtemanche et al., 1998). All of these would lead to uncertainties in the initial parameters and conditions due to experimental error and lack of knowledge. Likewise, the choice of the computational methods or resources used to perform the model parameterization and simulations can produce uncertainty in model results. This is because cardiac models may use different mathematical equations to describe the same physiological process, perhaps based on different analyses or assumptions on the physical-world process. Using more than one (e.g., cell) model to confirm predictions or validate the mechanistic understanding of a process is therefore a useful strategy (see for example Sarkar and Sobie, 2011; Sanchez et al., 2014; Lancaster and Sobie, 2016; Muszkiewicz et al., 2017). Additionally, even the choice of the numerical solver used by the software can lead to variability in model outputs, i.e., simulator uncertainty (Pathmanathan et al., 2012). Moreover, uncertainty in model outputs may arise if the code has not been verified to truly represent the mathematical equations in the computational model (Niederer et al., 2011a). Finally, optimization procedures can also introduce uncertainty, whereby the choice of whether to optimize simplified models with few parameters (Bueno-Orovio et al., 2008; Al Abed et al., 2013; Guo et al., 2013) or detailed models but only a few properties (e.g., focusing on specific currents) (Zhou et al., 2009; Fink et al., 2011) can lead to multiple distinct models given the same experimental data.

Used in conjunction with the approaches discussed in this review that take into account electrophysiological variability, new methods have been developed that try to quantify uncertainty more generally (Marino et al., 2008). Uncertainty quantification methods aim to quantify uncertainties in model inputs and propagation through the model to see how they affect model predictions (Pathmanathan et al., 2015; Mirams et al., 2016). This is typically done by assigning probability distribution functions, rather than fixed values to model parameters, as done for example by Pathmanathan et al. and applied to the study of I_Na_ steady-state inactivation (Pathmanathan et al., 2015). However, this process can be slow and tedious (requiring lots of simulations), especially if using a Monte Carlo sampling method that chooses input values from a statistical distribution. Also, in some cases, this statistical distribution of input parameters can be difficult to obtain or justify experimentally. To solve this issue, uncertainty quantification analysis has developed surrogate models or emulators (e.g., polynomial chaos expansions, and Gaussian process emulators; Chang et al., 2015), which are fast-running statistical approximations of the computational models and are quite powerful when fit to carefully constructed training data. Formal studies using uncertainty quantification in cardiac models are still limited, given the huge number of parameters in cardiac models, and may require the development of new methods or computational techniques (Johnstone et al., 2016).

### Potential covariance in ionic conductances challenges the current method of incorporating variability

Currently, populations of cardiac models and sample-specific models typically calibrate or fit to maximal conductance values or transport rates of channels or receptors, based on the observation that changes in expression levels of ion channels and transport proteins are the primary contributors to (inter-species) variability (Rosati et al., 2008). However, this approach does not take into account that the expression of ion channels will vary over relatively short time scales given changes in transcription, translation, degradation, or even circadian rhythm. Moreover, with a few exceptions (Sarkar and Sobie, 2011; Cummins et al., 2014) these methods do not typically account for variability in ion channel kinetics, which is known to change especially in response to drugs (Clancy et al., 2007). The methods discussed in this review can attempt to account for these properties using additional parameters.

Although the correlation between parameters (i.e., maximal conductances) is assessed sometimes (Britton et al., 2013), neither population-based nor the sample-specific approaches account for possible covariance in ion channel conductances, despite the fact it has been observed in neurons (Schulz et al., 2006, 2007; Tobin et al., 2009) and cardiac tissue (Schram et al., 2002; Rosati and Mckinnon, 2004; Deschenes et al., 2008; Xiao et al., 2008; Banyasz et al., 2011; Milstein et al., 2012). The exact mechanisms responsible for these covariances are still being explored. Xiao et al. found that sustained reductions in I_Kr_ may lead to compensatory upregulation of I_Ks_ through post-transcriptional upregulation of underlying subunits (Xiao et al., 2008), which potentially underlie the observed phenomenon of repolarization reserve (Roden, 2008). Macromolecular complexes or post-transcriptional modifications could also facilitate coregulation of ionic conductances, as demonstrated by the observed structural or functional complex between I_to_ and I_Na_ (Deschenes et al., 2008). Rees et al. recently argued that sensing of aggregate CaT may be sufficient in itself to regulate ionic conductances (of K^+^ and inward Ca^2+^) to maintain normal Ca^2+^ handling (Rees et al., 2018). Moreover, knockout and knockdown studies are consistent with the idea that cardiac cells have compensatory mechanisms to maintain AP or CaTs (perhaps to prevent arrhythmias) (Guo et al., 1999; Zhou et al., 2003). The covariance of ionic conductance can have significant implications for both calibrating populations of models or fitting sample-specific models, because it could propose additional constraints for how the underlying parameters of the computational model can be varied. Thus, new methods have begun to be developed that measure all ionic conductances at once, and can not only tease out how ionic conductances are correlated, but the extent to which they vary between cells (Banyasz et al., 2011; Groenendaal et al., 2015).

### Arrhythmia research requires understanding variability at larger spatial scales

Accounting for variability at tissue and organ-level scales is a logical, but not trivial (Elshrif and Cherry, 2014), next step. A thorough investigation of variability would first require including differences among the cells in the same tissue, and evaluating the impact of diverse geometrical distributions. One should also account for patient-specific structural differences, based on measures of tissue conductivity and anatomic properties, including heterogeneity in signaling due to non-uniform innervation. This last step can be particularly problematic when investigating diseased conditions affected by pronounced structural remodeling, such as fibrosis, organ dilation, and alterations in gap junction coupling. Where there has been meaningful progress in accounting for variability is in developing personalized atrial model structures based on medical images (Dossel et al., 2012; Trayanova, 2014). These methods have shown some promise in developing personalized ablation strategies (Mcdowell et al., 2015). For example, recently, Soor et al. implemented a modeling approach to optimize ablation times based on patient-specific atrial geometries to create lesions for a given atrial wall thickness (Soor et al., 2016). Combining these methods that utilize medical images with the methods described here, could significantly improve the clinical value of both methods alone (Hansen et al., 2017; Zhao et al., 2017). For example, Arevalo et al. developed personalized heart models based on cardiac imaging and published patch-clamp data to better predict arrhythmic events and possibly avoid unnecessary implantable cardioverter defibrillators (Arevalo et al., 2016). Developing multi-scale frameworks that account for variability is the next frontier in cardiac modeling that will greatly benefit from further advancements in computing capabilities. Beyond the availability of large experimental and clinical datasets, the development of novel techniques to speed model derivations and to integrate automation will be crucial to capture variability for different cell types and conditions at various scales.

### Safety pharmacology requires complementary electrophysiological experimental methods

The *in silico* approaches described here are being combined with other state-of-the-art tools to improve the evaluation of drug safety. Of significance, these approaches can help further the mission of the CiPA (Comprehensive *in vitro* Proarrhythmia Assay) initiative, which aspires to develop better methods to predict TdP. Beyond exclusively using steady-state hERG block as the main predictor of arrhythmia and not at all using QT interval prolongation, the CiPA initiative attempts to gain a more comprehensive understanding of proarrhythmic risk by combining (1) mechanistically-based *in vitro* assays, (2) *in silico* reconstructions of cardiac electrophysiology, and (3) confirmation using human iPSC-CMs (Colatsky et al., 2016). The methods described in this review are being utilized to help meet the mission of the CiPA initiative (Cummins et al., 2014; Lancaster and Sobie, 2016; Britton et al., 2017a; Passini et al., 2017). Most of the methods described here that assess the effects of drugs on populations of cardiac myocytes use simple pore block schemes. However, it is also clear that the sole use of steady-state hERG block assays is insufficient to predict arrhythmia risk, and thus studies are beginning to simulate the effects drug-binding kinetics and state-specific binding, which have been shown to affect electrophysiological outcomes (Lee W. et al., 2016; Dutta et al., 2017; Ellinwood et al., 2017b; Li et al., 2017). Incorporating more detailed drug-binding effects may allow studying the effects of populations of drugs characteristics (e.g., state-dependent block and kinetics) on populations of cardiomyocytes.

## Conclusion

Computational approaches that have been developed over the past decade to account for variability in cardiac electrophysiology have led to important insights into mechanisms of arrhythmogenesis, etiology of disease, and variable response to drugs. The approaches outlined in this review are used in basic research studies, i.e., quite separately from actual clinical workflows, where decisions are made sometimes for a particular patient within minutes. Advanced computing facilities now allow near real-time simulations of anatomically realistic, biophysically detailed models of human cardiac electrophysiology (Niederer et al., 2011b; Okada et al., 2015, 2017). Such massively parallel processes could be optimized to run personalized cardiac simulations pre-determined to have clinical value. However, implementing these approaches more comprehensively into clinical workflows still presents challenges and simulation of variability may not find immediate application.

## Author contributions

HN, SM, and EG reviewed the literature and wrote the manuscript.

### Conflict of interest statement

The authors declare that the research was conducted in the absence of any commercial or financial relationships that could be construed as a potential conflict of interest.

## Abbreviations

AF: atrial fibrillation
AP: action potential
APD: AP duration
cAF: chronic AF
CaT: Ca^2+^ transient
CiPA: Comprehensive *in vitro* Proarrhythmia Assay
DAD: delayed afterdepolarization
diLQTS: drug-induced long QT syndrome
EAD: early afterdepolarization
ECG: electrocardiogram
FRD: forward rate dependence
GA: genetic algorithm
hERG: human *ether-à-go-go*-related gene
hMSCs: human mesenchymal stem cells
I_CaL_: L-type Ca^2+^ current
I_to_: transient outward K^+^ current
I_Kur_: ultra-rapid delayed-rectifier K^+^ current
I_Kr_: rapid delayed-rectifier K^+^ current
I_Ks_: slow delayed-rectifier K^+^ current
I_K1_: inward rectifier K^+^ current
I_Na_: fast Na^+^ current
I_NaL_: late Na^+^ current
iPSC-CM: induced pluripotent stem-cell-derived cardiomyocyte
LHS: latin hypercube sampling
LQT3: long QT syndrome type 3
NCX: Na^+^/Ca^2+^ exchanger
nSR: normal sinus rhythm
SERCA: sarco/endoplasmic reticulum Ca^2+^-ATPase
TdP: torsades de pointes.

## References

[B1] Abramson D. Bernabeu M. O. Bethwaite B. Burrage K. Corrias A. Enticott C.. (2010). High-throughput cardiac science on the Grid. Philos. Trans. A Math. Phys. Eng. Sci. 368, 3907–3923. 10.1098/rsta.2010.0170 20643684

[B2] Achard P. De Schutter E. (2006). Complex parameter landscape for a complex neuron model. PLoS. Comput. Biol. 2:e94. 10.1371/journal.pcbi.0020094 16848639 PMC1513272

[B3] Adeniran I. El Harchi A. Hancox J. C. Zhang H. (2012). Proarrhythmia in KCNJ2-linked short QT syndrome: insights from modelling. Cardiovasc. Res. 94, 66–76. 10.1093/cvr/cvs082 22308236

[B4] Ahrens-Nicklas R. C. Clancy C. E. Christini D. J. (2009). Re-evaluating the efficacy of beta-adrenergic agonists and antagonists in long QT-3 syndrome through computational modelling. Cardiovasc. Res. 82, 439–447. 10.1093/cvr/cvp083 19264765 PMC2682617

[B5] Al Abed A. Guo T. Lovell N. H. Dokos S. (2013). Optimisation of ionic models to fit tissue action potentials: application to 3D atrial modelling. Comput. Math. Methods Med. 2013:951234 10.1155/2013/951234 23935704 PMC3713319

[B6] Arevalo H. J. Vadakkumpadan F. Guallar E. Jebb A. Malamas P. Wu K. C.. (2016). Arrhythmia risk stratification of patients after myocardial infarction using personalized heart models. Nat. Commun. 7:11437. 10.1038/ncomms11437 27164184 PMC4866040

[B7] Banyasz T. Horvath B. Jian Z. Izu L. T. Chen-Izu Y. (2011). Sequential dissection of multiple ionic currents in single cardiac myocytes under action potential-clamp. J. Mol. Cell. Cardiol. 50, 578–581. 10.1016/j.yjmcc.2010.12.020 21215755 PMC3047417

[B8] Barichello S. Roberts J. D. Backx P. Boyle P. M. Laksman Z. (2018). Personalizing therapy for atrial fibrillation: the role of stem cell and *in silico* disease models. Cardiovasc. Res. 114, 931–943. 10.1093/cvr/cvy090 29648634

[B9] Batchvarov V. N. Ghuran A. Smetana P. Hnatkova K. Harries M. Dilaveris P.. (2002). QT-RR relationship in healthy subjects exhibits substantial intersubject variability and high intrasubject stability. Am. J. Physiol. Heart Circ. Physiol. 282, H2356–H2363. 10.1152/ajpheart.00860.2001 12003846

[B10] Beattie K. A. Hill A. P. Bardenet R. Cui Y. Vandenberg J. I. Gavaghan D. J.. (2018). Sinusoidal voltage protocols for rapid characterisation of ion channel kinetics. J. Physiol. 596, 1813–1828. 10.1113/JP275733 29573276 PMC5978315

[B11] Beeler G. W. Reuter H. (1977). Reconstruction of the action potential of ventricular myocardial fibres. J. Physiol. 268, 177–210. 10.1113/jphysiol.1977.sp011853 874889 PMC1283659

[B12] Behar J. Yaniv Y. (2017). Age-related pacemaker deterioration is due to impaired intracellular and membrane mechanisms: insights from numerical modeling. J. Gen. Physiol. 149, 935–949. 10.1085/jgp.201711792 28887411 PMC5694941

[B13] Ben-Shalom R. Aviv A. Razon B. Korngreen A. (2012). Optimizing ion channel models using a parallel genetic algorithm on graphical processors. J. Neurosci. Methods 206, 183–194. 10.1016/j.jneumeth.2012.02.024 22407006

[B14] Bot C. T. Kherlopian A. R. Ortega F. A. Christini D. J. Krogh-Madsen T. (2012). Rapid genetic algorithm optimization of a mouse computational model: benefits for anthropomorphization of neonatal mouse cardiomyocytes. Front. Physiol. 3:421. 10.3389/fphys.2012.00421 23133423 PMC3488799

[B15] Boutjdir M. Le Heuzey J. Y. Lavergne T. Chauvaud S. Guize L. Carpentier A.. (1986). Inhomogeneity of cellular refractoriness in human atrium: factor of arrhythmia? Pacing Clin. Electrophysiol. 9, 1095–1100. 10.1111/j.1540-8159.1986.tb06676.x 2432515

[B16] Britton O. J. Abi-Gerges N. Page G. Ghetti A. Miller P. E. Rodriguez B. (2017a). Quantitative comparison of effects of dofetilide, sotalol, quinidine, and verapamil between human *ex vivo* trabeculae and *in silico* ventricular models incorporating inter-individual action potential variability. Front. Physiol. 8:597. 10.3389/fphys.2017.00597 28868038 PMC5563361

[B17] Britton O. J. Bueno-Orovio A. Van Ammel K. Lu H. R. Towart R. Gallacher D. J.. (2013). Experimentally calibrated population of models predicts and explains intersubject variability in cardiac cellular electrophysiology. Proc. Natl. Acad. Sci. U.S.A. 110, E2098–E2105. 10.1073/pnas.1304382110 23690584 PMC3677477

[B18] Britton O. J. Bueno-Orovio A. Virag L. Varro A. Rodriguez B. (2017b). The Electrogenic Na(+)/K(+) Pump is a key determinant of repolarization abnormality susceptibility in human ventricular cardiomyocytes: a population-based simulation study. Front. Physiol. 8:278 10.3389/fphys.2017.00278 28529489 PMC5418229

[B19] Bueno-Orovio A. Cherry E. M. Fenton F. H. (2008). Minimal model for human ventricular action potentials in tissue. J. Theor. Biol. 253, 544–560. 10.1016/j.jtbi.2008.03.029 18495166

[B20] Bueno-Orovio A. Sánchez C. Pueyo E. Rodriguez B. (2014). Na/K pump regulation of cardiac repolarization: insights from a systems biology approach. Pflugers Arch. 466, 183–193. 10.1007/s00424-013-1293-1 23674099

[B21] Burrage K. Burrage P. Donovan D. Thompson B. (2015). Populations of models, experimental designs and coverage of parameter space by latin hypercube and orthogonal sampling. Procedia Comp. Sci. 51, 1762–1771. 10.1016/j.procs.2015.05.383

[B22] Butters T. D. Aslanidi O. V. Inada S. Boyett M. R. Hancox J. C. Lei M.. (2010). Mechanistic links between Na+ channel (SCN5A) mutations and impaired cardiac pacemaking in sick sinus syndrome. Circ. Res. 107, 126–137. 10.1161/CIRCRESAHA.110.219949 20448214 PMC2901593

[B23] Campbell S. G. Flaim S. N. Leem C. H. Mcculloch A. D. (2008). Mechanisms of transmurally varying myocyte electromechanics in an integrated computational model. Philos. Trans. A Math. Phys. Eng. Sci. 366, 3361–3380. 10.1098/rsta.2008.0088 18593662 PMC2556206

[B24] Chang E. T. Strong M. Clayton R. H. (2015). Bayesian sensitivity analysis of a cardiac cell model using a gaussian process emulator. PLoS ONE 10:e0130252 10.1371/journal.pone.0130252 26114610 PMC4482712

[B25] Chen Q. Guan S. U. (2004). Incremental multiple objective genetic algorithms. IEEE. Trans. Syst. Man. Cybern. B Cybern. 34, 1325–1334. 10.1109/TSMCB.2003.822958 15484906

[B26] Clancy C. E. Rudy Y. (1999). Linking a genetic defect to its cellular phenotype in a cardiac arrhythmia. Nature 400, 566–569. 10.1038/23034 10448858

[B27] Clancy C. E. Rudy Y. (2002). Na(+) channel mutation that causes both Brugada and long-QT syndrome phenotypes: a simulation study of mechanism. Circulation 105, 1208–1213. 10.1161/hc1002.105183 11889015 PMC1997279

[B28] Clancy C. E. Tateyama M. Kass R. S. (2002). Insights into the molecular mechanisms of bradycardia-triggered arrhythmias in long QT-3 syndrome. J. Clin. Invest. 110, 1251–1262. 10.1172/JCI0215928 12417563 PMC151612

[B29] Clancy C. E. Zhu Z. I. Rudy Y. (2007). Pharmacogenetics and anti-arrhythmic drug therapy: a theoretical investigation. Am. J. Physiol. Heart Circ. Physiol. 292, H66–H75. 10.1152/ajpheart.00312.2006 16997895 PMC2034498

[B30] Colatsky T. Fermini B. Gintant G. Pierson J. B. Sager P. Sekino Y.. (2016). The Comprehensive *in Vitro* Proarrhythmia Assay (CiPA) initiative - Update on progress. J. Pharmacol. Toxicol. Methods 81, 15–20. 10.1016/j.vascn.2016.06.002 27282641

[B31] Colman M. A. Aslanidi O. V. Kharche S. Boyett M. R. Garratt C. Hancox J. C.. (2013). Pro-arrhythmogenic effects of atrial fibrillation-induced electrical remodelling: insights from the three-dimensional virtual human atria. J. Physiol. 591, 4249–4272. 10.1113/jphysiol.2013.254987 23732649 PMC3779115

[B32] Comtois P. Sakabe M. Vigmond E. J. Munoz M. Texier A. Shiroshita-Takeshita A.. (2008). Mechanisms of atrial fibrillation termination by rapidly unbinding Na+ channel blockers: insights from mathematical models and experimental correlates. Am. J. Physiol. Heart. Circ. Physiol. 295, H1489–H1504. 10.1152/ajpheart.01054.2007 18676686

[B33] Corrias A. Giles W. Rodriguez B. (2011). Ionic mechanisms of electrophysiological properties and repolarization abnormalities in rabbit Purkinje fibers. Am. J. Physiol. Heart Circ. Physiol. 300, H1806–H1813. 10.1152/ajpheart.01170.2010 21335469

[B34] Courtemanche M. Ramirez R. J. Nattel S. (1998). Ionic mechanisms underlying human atrial action potential properties: insights from a mathematical model. Am. J. Physiol. 275, H301–H321. 10.1152/ajpheart.1998.275.1.H301 9688927

[B35] Courtemanche M. Winfree A. T. (1991). Re-entrant rotating waves in a beeler-reuter based model of two-dimensional cardiac electrical activity. Int. J. Bifurcation Chaos 1, 431–444. 10.1142/S0218127491000336

[B36] Crosby J. L. (1982). Computer Simulation in Genetics. London, New York, NY: John Wiley.

[B37] Cummins M. A. Dalal P. J. Bugana M. Severi S. Sobie E. A. (2014). Comprehensive analyses of ventricular myocyte models identify targets exhibiting favorable rate dependence. PLoS. Comput. Biol. 10:e1003543. 10.1371/journal.pcbi.1003543 24675446 PMC3967944

[B38] Daniels G. S. (1952). The Average Man? Technical Note WCRD53-7, Wright Air Development Center, Ohio AD-10203.

[B39] Deng D. Murphy M. J. Hakim J. B. Franceschi W. H. Zahid S. Pashakhanloo F.. (2017). Sensitivity of reentrant driver localization to electrophysiological parameter variability in image-based computational models of persistent atrial fibrillation sustained by a fibrotic substrate. Chaos 27, 093932. 10.1063/1.5003340 28964164 PMC5605332

[B40] Deschênes I. Armoundas A. A. Jones S. P. Tomaselli G. F. (2008). Post-transcriptional gene silencing of KChIP2 and Navbeta1 in neonatal rat cardiac myocytes reveals a functional association between Na and Ito currents. J. Mol. Cell. Cardiol. 45, 336–346. 10.1016/j.yjmcc.2008.05.001 18565539 PMC2580777

[B41] Devenyi R. A. Ortega F. A. Groenendaal W. Krogh-Madsen T. Christini D. J. Sobie E. A. (2017). Differential roles of two delayed rectifier potassium currents in regulation of ventricular action potential duration and arrhythmia susceptibility. J. Physiol. 595, 2301–2317. 10.1113/JP273191 27779762 PMC5374112

[B42] Devenyi R. A. Sobie E. A. (2016). There and back again: iterating between population-based modeling and experiments reveals surprising regulation of calcium transients in rat cardiac myocytes. J. Mol. Cell. Cardiol. 96, 38–48. 10.1016/j.yjmcc.2015.07.016 26235057 PMC4733425

[B43] Dibb K. Trafford A. Zhang H. Eisner D. (2015). A model model: a commentary on DiFrancesco and Noble (1985) 'A model of cardiac electrical activity incorporating ionic pumps and concentration changes'. Philos. Trans. R Soc. Lond. B Biol. Sci. 370:20140316 10.1098/rstb.2014.0316 25750236 PMC4360121

[B44] Difrancesco D. Noble D. (1985). A model of cardiac electrical activity incorporating ionic pumps and concentration changes. Philos. Trans. R Soc. Lond. B Biol. Sci. 307, 353–398. 10.1098/rstb.1985.0001 2578676

[B45] Dokos S. Lovell N. H. (2004). Parameter estimation in cardiac ionic models. Prog. Biophys. Mol. Biol. 85, 407–431. 10.1016/j.pbiomolbio.2004.02.002 15142755

[B46] Donovan D. Burrage K. Burrage P. Mccourt T. A. Thompson B. Yazici E. S. (2018). Estimates of the coverage of parameter space by latin hypercube and orthogonal array-based sampling. Appl. Math. Model. 57, 553–564. 10.1016/j.apm.2017.11.036

[B47] Dössel O. Krueger M. W. Weber F. M. Wilhelms M. Seemann G. (2012). Computational modeling of the human atrial anatomy and electrophysiology. Med. Biol. Eng. Comput. 50, 773–799. 10.1007/s11517-012-0924-6 22718317

[B48] Draper N. R. Smith H. (2014). Applied Regression Analysis. Hoboken; Somerset, NJ: John Wiley and Sons.

[B49] Druckmann S. Banitt Y. Gidon A. Schürmann F. Markram H. Segev I. (2007). A novel multiple objective optimization framework for constraining conductance-based neuron models by experimental data. Front. Neurosci. 1, 7–18. 10.3389/neuro.01.1.1.001.2007 18982116 PMC2570085

[B50] Dutta S. Chang K. C. Beattie K. A. Sheng J. Tran P. N. Wu W. W. (2017). Optimization of an *in silico* cardiac cell model for proarrhythmia risk assessment. Front. Physiol. 8:616 10.3389/fphys.2017.00616 28878692 PMC5572155

[B51] Edwards A. G. Grandi E. Hake J. E. Patel S. Li P. Miyamoto S.. (2014). Nonequilibrium reactivation of Na+ current drives early afterdepolarizations in mouse ventricle. Circ. Arrhythm Electrophysiol. 7, 1205–1213. 10.1161/CIRCEP.113.001666 25236710 PMC4301603

[B52] Ellinwood N. Dobrev D. Morotti S. Grandi E. (2017a). *In silico* assessment of efficacy and safety of IKur inhibitors in chronic atrial fibrillation: role of kinetics and state-dependence of drug binding. Front. Pharmacol. 8, 799. 10.3389/fphar.2017.00799 29163179 PMC5681918

[B53] Ellinwood N. Dobrev D. Morotti S. Grandi E. (2017b). Revealing kinetics and state-dependent binding properties of IKur-targeting drugs that maximize atrial fibrillation selectivity. Chaos 27, 093918 10.1063/1.5000226 28964116 PMC5573366

[B54] Elshrif M. M. Cherry E. M. (2014). A quantitative comparison of the behavior of human ventricular cardiac electrophysiology models in tissue. PLoS ONE 9:e84401. 10.1371/journal.pone.0084401 24416228 PMC3885549

[B55] Fender E. A. Henrikson C. A. Tereshchenko L. (2014). Racial differences in sudden cardiac death. J. Electrocardiol. 47, 815–818. 10.1016/j.jelectrocard.2014.07.023 25155390 PMC4252611

[B56] Feng J. Yue L. Wang Z. Nattel S. (1998). Ionic mechanisms of regional action potential heterogeneity in the canine right atrium. Circ. Res. 83, 541–551. 10.1161/01.RES.83.5.541 9734477

[B57] Fink M. Niederer S. A. Cherry E. M. Fenton F. H. Koivumäki J. T. Seemann G.. (2011). Cardiac cell modelling: observations from the heart of the cardiac physiome project. Prog. Biophys. Mol. Biol. 104, 2–21. 10.1016/j.pbiomolbio.2010.03.002 20303361

[B58] Fotiadis P. Forger D. B. (2013). Modeling the effects of the circadian clock on cardiac electrophysiology. J. Biol. Rhythms 28, 69–78. 10.1177/0748730412469499 23382593

[B59] Fox J. J. Mcharg J. L. Gilmour R. F. (2002). Ionic mechanism of electrical alternans. Am. J. Physiol. Heart Circ. Physiol. 282, H516–H530. 10.1152/ajpheart.00612.2001 11788399

[B60] Fraser A. Burnell D. (1970). Computer Models in Genetics. New York, NY: McGraw-Hill.

[B61] Gaborit N. Le Bouter S. Szuts V. Varro A. Escande D. Nattel S.. (2007). Regional and tissue specific transcript signatures of ion channel genes in the non-diseased human heart. J. Physiol. 582, 675–693. 10.1113/jphysiol.2006.126714 17478540 PMC2075332

[B62] Gaspo R. Bosch R. F. Talajic M. Nattel S. (1997). Functional mechanisms underlying tachycardia-induced sustained atrial fibrillation in a chronic dog model. Circulation 96, 4027–4035. 10.1161/01.CIR.96.11.4027 9403628

[B63] Geladi P. Kowalski B. R. (1986). Partial least-squares regression - a tutorial. Anal. Chim. Acta 185, 1–17. 10.1016/0003-2670(86)80028-9

[B64] Golowasch J. Goldman M. S. Abbott L. F. Marder E. (2002). Failure of averaging in the construction of a conductance-based neuron model. J. Neurophys. 87, 1129–1131. 10.1152/jn.00412.2001 11826077

[B65] Gong J. Q. X. Sobie E. A. (2018). Population-based mechanistic modeling allows for quantitative predictions of drug responses across cell types. NPJ. Syst. Biol. Appl. 4, 11. 10.1038/s41540-018-0047-2 29507757 PMC5825396

[B66] Gong Y. Xie F. Stein K. M. Garfinkel A. Culianu C. A. Lerman B. B.. (2007). Mechanism underlying initiation of paroxysmal atrial flutter/atrial fibrillation by ectopic foci: a simulation study. Circulation 115, 2094–2102. 10.1161/CIRCULATIONAHA.106.656504 17420354

[B67] Grandi E. Maleckar M. M. (2016). Anti-arrhythmic strategies for atrial fibrillation: the role of computational modeling in discovery, development, and optimization. Pharmacol. Ther. 168, 126–142. 10.1016/j.pharmthera.2016.09.012 27612549 PMC5140742

[B68] Grandi E. Pandit S. V. Voigt N. Workman A. J. Dobrev D. Jalife J.. (2011). Human atrial action potential and Ca^2+^ model: sinus rhythm and chronic atrial fibrillation. Circ. Res. 109, 1055–1066. 10.1161/CIRCRESAHA.111.253955 21921263 PMC3208665

[B69] Grandi E. Pasqualini F. S. Bers D. M. (2010). A novel computational model of the human ventricular action potential and Ca transient. J. Mol. Cell. Cardiol. 48, 112–121. 10.1016/j.yjmcc.2009.09.019 19835882 PMC2813400

[B70] Gray R. A. Jalife J. Panfilov A. Baxter W. T. Cabo C. Davidenko J. M.. (1995). Nonstationary vortexlike reentrant activity as a mechanism of polymorphic ventricular tachycardia in the isolated rabbit heart. Circulation 91, 2454–2469. 10.1161/01.CIR.91.9.2454 7729033

[B71] Groenendaal W. Ortega F. A. Kherlopian A. R. Zygmunt A. C. Krogh-Madsen T. Christini D. J. (2015). Cell-specific cardiac electrophysiology models. PLoS Comput. Biol. 11:e1004242. 10.1371/journal.pcbi.1004242 25928268 PMC4415772

[B72] Guo T. Abed A. A. Lovell N. H. Dokos S. (2010). A generic ionic model of cardiac action potentials. Conf. Proc. IEEE. Eng. Med. Biol. Soc. 2010, 1465–1468. 10.1109/IEMBS.2010.5626853 21096358

[B73] Guo T. Al Abed A. Lovell N. H. Dokos S. (2013). Optimisation of a generic ionic model of cardiac myocyte electrical activity. Comput. Math. Methods Med. 2013:706195 10.1155/2013/706195 23710254 PMC3659483

[B74] Guo W. Xu H. London B. Nerbonne J. M. (1999). Molecular basis of transient outward K+ current diversity in mouse ventricular myocytes. J. Physiol. 521(Pt 3), 587–599. 10.1111/j.1469-7793.1999.00587.x 10601491 PMC2269690

[B75] Gurkiewicz M. Korngreen A. (2007). A numerical approach to ion channel modelling using whole-cell voltage-clamp recordings and a genetic algorithm. PLoS Comput. Biol. 3:e169. 10.1371/journal.pcbi.0030169 17784781 PMC1963494

[B76] Hair J. F. Black W. C. Babin B. J. Anderson R. E. (2010). Multivariate Data Analysis. Upper Saddle River, NJ: Pearson Prentice Hall.

[B77] Hansen B. J. Zhao J. Fedorov V. V. (2017). Fibrosis and atrial fibrillation: computerized and optical mapping; a view into the human atria at submillimeter resolution. JACC Clin. Electrophysiol. 3, 531–546. 10.1016/j.jacep.2017.05.002 29159313 PMC5693365

[B78] Heijman J. Zaza A. Johnson D. M. Rudy Y. Peeters R. L. Volders P. G.. (2013). Determinants of beat-to-beat variability of repolarization duration in the canine ventricular myocyte: a computational analysis. PLoS Comput. Biol. 9:e1003202. 10.1371/journal.pcbi.1003202 23990775 PMC3749940

[B79] Hobbs K. H. Hooper S. L. (2008). Using complicated, wide dynamic range driving to develop models of single neurons in single recording sessions. J. Neurophysiol. 99, 1871–1883. 10.1152/jn.00032.2008 18256169

[B80] Hodgkin A. L. Huxley A. F. (1952). A quantitative description of membrane current and its application to conduction and excitation in nerve. J. Physiol. 117, 500–544. 10.1113/jphysiol.1952.sp004764 12991237 PMC1392413

[B81] Hund T. J. Rudy Y. (2004). Rate dependence and regulation of action potential and calcium transient in a canine cardiac ventricular cell model. Circulation 110, 3168–3174. 10.1161/01.CIR.0000147231.69595.D3 15505083 PMC1851913

[B82] Hussein Y. A. El-Ghazaly S. M. (2004). Modeling and optimization of microwave devices and circuits using genetic algorithms. IEEE Trans. Microwave Theory Tech. 52, 329–336. 10.1109/TMTT.2003.820899

[B83] Inada S. Hancox J. C. Zhang H. Boyett M. R. (2009). One-dimensional mathematical model of the atrioventricular node including atrio-nodal, nodal, and nodal-his cells. Biophys. J. 97, 2117–2127. 10.1016/j.bpj.2009.06.056 19843444 PMC2764063

[B84] Janes K. A. Albeck J. G. Gaudet S. Sorger P. K. Lauffenburger D. A. Yaffe M. B. (2005). A systems model of signaling identifies a molecular basis set for cytokine-induced apoptosis. Science 310, 1646–1653. 10.1126/science.1116598 16339439

[B85] Jeyaraj D. Haldar S. M. Wan X. Mccauley M. D. Ripperger J. A. Hu K.. (2012). Circadian rhythms govern cardiac repolarization and arrhythmogenesis. Nature 483, 96–99. 10.1038/nature10852 22367544 PMC3297978

[B86] Johnstone R. H. Chang E. T. Y. Bardenet R. de Boer T. P. Gavaghan D. J. Pathmanathan P.. (2016). Uncertainty and variability in models of the cardiac action potential: can we build trustworthy models? J. Mol. Cell. Cardiol. 96, 49–62. 10.1016/j.yjmcc.2015.11.018 26611884 PMC4915860

[B87] Kannankeril P. J. Norris K. J. Carter S. Roden D. M. (2011). Factors affecting the degree of QT prolongation with drug challenge in a large cohort of normal volunteers. Heart Rhythm 8, 1530–1534. 10.1016/j.hrthm.2011.03.042 21420510 PMC3154568

[B88] Kaur J. Nygren A. Vigmond E. J. (2014). Fitting membrane resistance along with action potential shape in cardiac myocytes improves convergence: application of a multi-objective parallel genetic algorithm. PLoS ONE 9:e107984. 10.1371/journal.pone.0107984 25250956 PMC4176019

[B89] Kharche S. Garratt C. J. Boyett M. R. Inada S. Holden A. V. Hancox J. C.. (2008). Atrial proarrhythmia due to increased inward rectifier current (I(K1)) arising from KCNJ2 mutation–a simulation study. Prog. Biophys. Mol. Biol. 98, 186–197. 10.1016/j.pbiomolbio.2008.10.010 19041665

[B90] Koivumäki J. T. Naumenko N. Tuomainen T. Takalo J. Oksanen M. Puttonen K. A.. (2018). Structural immaturity of human iPSC-Derived cardiomyocytes: *in silico* investigation of effects on function and disease modeling. Front. Physiol. 9:80. 10.3389/fphys.2018.00080 29467678 PMC5808345

[B91] Krogh-Madsen T. Christini D. J. (2012). Nonlinear dynamics in cardiology. Annu. Rev. Biomed. Eng. 14, 179–203. 10.1146/annurev-bioeng-071811-150106 22524390 PMC3733460

[B92] Krogh-Madsen T. Jacobson A. F. Ortega F. A. Christini D. J. (2017). Global optimization of ventricular myocyte model to multi-variable objective improves predictions of drug-induced torsades de pointes. Front. Physiol. 8:1059. 10.3389/fphys.2017.01059 29311985 PMC5742183

[B93] Krogh-Madsen T. Sobie E. A. Christini D. J. (2016). Improving cardiomyocyte model fidelity and utility via dynamic electrophysiology protocols and optimization algorithms. J. Physiol. 594, 2525–2536. 10.1113/JP270618 26661516 PMC4850194

[B94] Kurata Y. Hisatome I. Matsuda H. Shibamoto T. (2005). Dynamical mechanisms of pacemaker generation in IK1-downregulated human ventricular myocytes: insights from bifurcation analyses of a mathematical model. Biophys. J. 89, 2865–2887. 10.1529/biophysj.105.060830 16040746 PMC1366784

[B95] Lancaster M. C. Sobie E. A. (2016). Improved prediction of drug-induced torsades de pointes through simulations of dynamics and machine learning algorithms. Clin. Pharmacol. Ther. 100, 371–379. 10.1002/cpt.367 26950176 PMC6375298

[B96] Lau C. P. Tse H. F. Siu C. W. Gbadebo D. (2012). Atrial electrical and structural remodeling: implications for racial differences in atrial fibrillation. J. Cardiovasc. Electrophysiol. 23(Suppl. 1), S36–S40. 10.1111/jce.12022 23140346

[B97] Lawson B. A. J. Drovandi C. C. Cusimano N. Burrage P. Rodriguez B. Burrage K. (2018). Unlocking data sets by calibrating populations of models to data density: a study in atrial electrophysiology. Sci. Adv. 4:e1701676. 10.1126/sciadv.1701676 29349296 PMC5770172

[B98] Lee W. Mann S. A. Windley M. J. Imtiaz M. S. Vandenberg J. I. Hill A. P. (2016). *In silico* assessment of kinetics and state dependent binding properties of drugs causing acquired LQTS. Prog. Biophys. Mol. Biol. 120, 89–99. 10.1016/j.pbiomolbio.2015.12.005 26713558

[B99] Lee Y. S. Hwang M. Song J. S. Li C. Joung B. Sobie E. A.. (2016). The contribution of ionic currents to rate-dependent action potential duration and pattern of reentry in a mathematical model of human atrial fibrillation. PLoS ONE 11:e0150779. 10.1371/journal.pone.0150779 26964092 PMC4795605

[B100] Lee Y. S. Liu O. Z. Hwang H. S. Knollmann B. C. Sobie E. A. (2013). Parameter sensitivity analysis of stochastic models provides insights into cardiac calcium sparks. Biophys. J. 104, 1142–1150. 10.1016/j.bpj.2012.12.055 23473497 PMC3870797

[B101] Leung F. H. F. Lam H. K. Ling S. H. Tam P. K. S. (2004). Optimal and stable fuzzy controllers for nonlinear systems based on an improved genetic algorithm. IEEE Trans. Indus. Electron. 51, 172–182. 10.1109/TIE.2003.821898

[B102] Li Z. Dutta S. Sheng J. Tran P. N. Wu W. Chang K.. (2017). Improving the *in silico* assessment of proarrhythmia risk by combining herg (human ether-a-go-go-related gene) channel-drug binding kinetics and multichannel pharmacology. Circ. Arrhythm. Electrophysiol. 10:e004628. 10.1161/CIRCEP.116.004628 28202629

[B103] Liberos A. Bueno-Orovio A. Rodrigo M. Ravens U. Hernandez-Romero I. Fernandez-Aviles F.. (2016). Balance between sodium and calcium currents underlying chronic atrial fibrillation termination: an *in silico* intersubject variability study. Heart Rhythm 13, 2358–2365. 10.1016/j.hrthm.2016.08.028 27569443 PMC5221730

[B104] Liu L. Nattel S. (1997). Differing sympathetic and vagal effects on atrial fibrillation in dogs: role of refractoriness heterogeneity. Am. J. Physiol. 273, H805–H816. 10.1152/ajpheart.1997.273.2.H805 9277498

[B105] Livshitz L. Rudy Y. (2009). Uniqueness and stability of action potential models during rest, pacing, and conduction using problem-solving environment. Biophys. J. 97, 1265–1276. 10.1016/j.bpj.2009.05.062 19720014 PMC2749757

[B106] Lombardo D. M. Fenton F. H. Narayan S. M. Rappel W. J. (2016). Comparison of detailed and simplified models of human atrial myocytes to recapitulate patient specific properties. PLoS Comput. Biol. 12:e1005060. 10.1371/journal.pcbi.1005060 27494252 PMC4975409

[B107] Luo C. H. Rudy Y. (1991). A model of the ventricular cardiac action potential. Depolarization, repolarization, and their interaction. Circ. Res. 68, 1501–1526. 10.1161/01.RES.68.6.1501 1709839

[B108] Maleckar M. M. Greenstein J. L. Giles W. R. Trayanova N. A. (2009). K+ current changes account for the rate dependence of the action potential in the human atrial myocyte. Am. J. Physiol. Heart Circ. Physiol. 297, H1398–H1410. 10.1152/ajpheart.00411.2009 19633207 PMC2770776

[B109] Maltsev V. A. Lakatta E. G. (2009). Synergism of coupled subsarcolemmal Ca2+ clocks and sarcolemmal voltage clocks confers robust and flexible pacemaker function in a novel pacemaker cell model. Am. J. Physiol. Heart Circ. Physiol. 296, H594–H615. 10.1152/ajpheart.01118.2008 19136600 PMC2660239

[B110] Mann S. A. Imtiaz M. Winbo A. Rydberg A. Perry M. D. Couderc J. P.. (2016). Convergence of models of human ventricular myocyte electrophysiology after global optimization to recapitulate clinical long QT phenotypes. J. Mol. Cell. Cardiol. 100, 25–34. 10.1016/j.yjmcc.2016.09.011 27663173

[B111] Mann S. A. Otway R. Guo G. Soka M. Karlsdotter L. Trivedi G.. (2012). Epistatic effects of potassium channel variation on cardiac repolarization and atrial fibrillation risk. J. Am. Coll. Cardiol. 59, 1017–1025. 10.1016/j.jacc.2011.11.039 22402074

[B112] Marder E. (2011). Variability, compensation, and modulation in neurons and circuits. Proc. Natl. Acad. Sci. U.S.A. 108(Suppl. 3), 15542–15548. 10.1073/pnas.1010674108 21383190 PMC3176600

[B113] Marino S. Hogue I. B. Ray C. J. Kirschner D. E. (2008). A methodology for performing global uncertainty and sensitivity analysis in systems biology. J. Theor. Biol. 254, 178–196. 10.1016/j.jtbi.2008.04.011 18572196 PMC2570191

[B114] Mayourian J. Cashman T. J. Ceholski D. K. Johnson B. V. Sachs D. Kaji D. A.. (2017). Experimental and computational insight into human mesenchymal stem cell paracrine signaling and heterocellular coupling effects on cardiac contractility and arrhythmogenicity. Circ. Res. 121, 411–423. 10.1161/CIRCRESAHA.117.310796 28642329 PMC5899516

[B115] McDowell K. S. Zahid S. Vadakkumpadan F. Blauer J. Macleod R. S. Trayanova N. A. (2015). Virtual electrophysiological study of atrial fibrillation in fibrotic remodeling. PLoS ONE 10:e0117110. 10.1371/journal.pone.0117110 25692857 PMC4333565

[B116] Milstein M. L. Musa H. Balbuena D. P. Anumonwo J. M. Auerbach D. S. Furspan P. B.. (2012). Dynamic reciprocity of sodium and potassium channel expression in a macromolecular complex controls cardiac excitability and arrhythmia. Proc. Natl. Acad. Sci. U.S.A. 109, E2134–E2143. 10.1073/pnas.1109370109 22509027 PMC3412015

[B117] Mirams G. R. Pathmanathan P. Gray R. A. Challenor P. Clayton R. H. (2016). Uncertainty and variability in computational and mathematical models of cardiac physiology. J. Physiol. 594, 6833–6847. 10.1113/JP271671 26990229 PMC5134370

[B118] Misier A. R. Opthof T. Van Hemel N. M. Defauw J. J. de Bakker J. M. Janse M. J.. (1992). Increased dispersion of “refractoriness” in patients with idiopathic paroxysmal atrial fibrillation. J. Am. Coll. Cardiol. 19, 1531–1535. 10.1016/0735-1097(92)90614-S 1593049

[B119] Moe G. K. Rheinboldt W. C. Abildskov J. A. (1964). A computer model of atrial fibrillation. Am. Heart J. 67, 200–220. 10.1016/0002-8703(64)90371-0 14118488

[B120] Morotti S. Grandi E. (2017). Logistic regression analysis of populations of electrophysiological models to assess proarrythmic risk. MethodsX 4, 25–34. 10.1016/j.mex.2016.12.002 28116246 PMC5225282

[B121] Morotti S. Mcculloch A. D. Bers D. M. Edwards A. G. Grandi E. (2016). Atrial-selective targeting of arrhythmogenic phase-3 early afterdepolarizations in human myocytes. J. Mol. Cell. Cardiol. 96, 63–71. 10.1016/j.yjmcc.2015.07.030 26241847 PMC4734906

[B122] Muszkiewicz A. Britton O. J. Gemmell P. Passini E. Sánchez C. Zhou X.. (2016). Variability in cardiac electrophysiology: using experimentally-calibrated populations of models to move beyond the single virtual physiological human paradigm. Prog. Biophys. Mol. Biol. 120, 115–127. 10.1016/j.pbiomolbio.2015.12.002 26701222 PMC4821179

[B123] Muszkiewicz A. Liu X. Bueno-Orovio A. Lawson B. A. J. Burrage K. Casadei B.. (2017). From ionic to cellular variability in human atrial myocytes: an integrative computational and experimental study. Am. J. Physiol. Heart Circ. Physiol. 314, H895–H916. 10.1152/ajpheart.00477.2017 29351467 PMC6008144

[B124] Ni H. Whittaker D. G. Wang W. Giles W. R. Narayan S. M. Zhang H. (2017). Synergistic anti-arrhythmic effects in human atria with combined use of sodium blockers and acacetin. Front. Physiol. 8:946. 10.3389/fphys.2017.00946 29218016 PMC5703742

[B125] Niederer S. A. Fink M. Noble D. Smith N. P. (2009). A meta-analysis of cardiac electrophysiology computational models. Exp. Physiol. 94, 486–495. 10.1113/expphysiol.2008.044610 19139063

[B126] Niederer S. A. Kerfoot E. Benson A. P. Bernabeu M. O. Bernus O. Bradley C.. (2011a). Verification of cardiac tissue electrophysiology simulators using an N-version benchmark. Philos. Trans. R. Soc. Math. Phys. Eng. Sci. 369, 4331–4351. 10.1098/rsta.2011.0139 21969679 PMC3263775

[B127] Niederer S. Mitchell L. Smith N. Plank G. (2011b). Simulating human cardiac electrophysiology on clinical time-scales. Front. Physiol. 2:14. 10.3389/fphys.2011.00014 21516246 PMC3079856

[B128] Noble D. (1962). A modification of the Hodgkin–Huxley equations applicable to Purkinje fibre action and pace-maker potentials. J. Physiol. 160, 317–352. 10.1113/jphysiol.1962.sp006849 14480151 PMC1359535

[B129] Noble D. Garny A. Noble P. J. (2012). How the Hodgkin-Huxley equations inspired the Cardiac Physiome Project. J. Physiol. Lond. 590, 2613–2628. 10.1113/jphysiol.2011.224238 22473779 PMC3424720

[B130] Noble D. Sarai N. Noble P. J. Kobayashi T. Matsuoka S. Noma A. (2007). Resistance of cardiac cells to NCX knockout: a model study. Ann. N. Y. Acad. Sci. 1099, 306–309. 10.1196/annals.1387.018 17446471

[B131] Nygren A. Fiset C. Firek L. Clark J. W. Lindblad D. S. Clark R. B.. (1998). Mathematical model of an adult human atrial cell: the role of K+ currents in repolarization. Circ. Res. 82, 63–81. 10.1161/01.RES.82.1.63 9440706

[B132] O'Hara T. Virág L. Varró A. Rudy Y. (2011). Simulation of the undiseased human cardiac ventricular action potential: model formulation and experimental validation. PLoS Comput. Biol. 7:e1002061. 10.1371/journal.pcbi.1002061 21637795 PMC3102752

[B133] Okada J. I. Washio T. Nakagawa M. Watanabe M. Kadooka Y. Kariya T.. (2017). Multi-scale, tailor-made heart simulation can predict the effect of cardiac resynchronization therapy. J. Mol. Cell. Cardiol. 108, 17–23. 10.1016/j.yjmcc.2017.05.006 28502795

[B134] Okada J. Yoshinaga T. Kurokawa J. Washio T. Furukawa T. Sawada K.. (2015). Screening system for drug-induced arrhythmogenic risk combining a patch clamp and heart simulator. Sci. Adv. 1:e1400142. 10.1126/sciadv.1400142 26601174 PMC4640654

[B135] Paci M. Passini E. Severi S. Hyttinen J. Rodriguez B. (2017). Phenotypic variability in LQT3 human induced pluripotent stem cell-derived cardiomyocytes and their response to antiarrhythmic pharmacologic therapy: an *in silico* approach. Heart Rhythm 14, 1704–1712. 10.1016/j.hrthm.2017.07.026 28756098 PMC5668441

[B136] Pandit S. V. Clark R. B. Giles W. R. Demir S. S. (2001). A mathematical model of action potential heterogeneity in adult rat left ventricular myocytes. Biophys. J. 81, 3029–3051. 10.1016/S0006-3495(01)75943-7 11720973 PMC1301767

[B137] Panfilov A. V. Holden A. V. (1991). Spatiotemporal irregularity in a two-dimensional model of cardiac tissue. Int. J. Bifurcation Chaos 1, 219–225. 10.1142/S0218127491000142

[B138] Passini E. Britton O. J. Lu H. R. Rohrbacher J. Hermans A. N. Gallacher D. J.. (2017). Human *in silico* drug trials demonstrate higher accuracy than animal models in predicting clinical pro-arrhythmic cardiotoxicity. Front. Physiol. 8:668. 10.3389/fphys.2017.00668 28955244 PMC5601077

[B139] Passini E. Mincholé A. Coppini R. Cerbai E. Rodriguez B. Severi S.. (2016). Mechanisms of pro-arrhythmic abnormalities in ventricular repolarisation and anti-arrhythmic therapies in human hypertrophic cardiomyopathy. J. Mol. Cell. Cardiol. 96, 72–81. 10.1016/j.yjmcc.2015.09.003 26385634 PMC4915817

[B140] Pathmanathan P. Bernabeu M. O. Niederer S. A. Gavaghan D. J. Kay D. (2012). Computational modelling of cardiac electrophysiology: explanation of the variability of results from different numerical solvers. Int. J. Numer. Methods Biomed. Eng. 28, 890–903. 10.1002/cnm.2467 25099569

[B141] Pathmanathan P. Shotwell M. S. Gavaghan D. J. Cordeiro J. M. Gray R. A. (2015). Uncertainty quantification of fast sodium current steady-state inactivation for multi-scale models of cardiac electrophysiology. Prog. Biophys. Mol. Biol. 117, 4–18. 10.1016/j.pbiomolbio.2015.01.008 25661325 PMC4472478

[B142] Pitt-Francis J. Garny A. Gavaghan D. (2006). Enabling computer models of the heart for high-performance computers and the grid. Philos. Trans. A Math. Phys. Eng. Sci. 364, 1501–1516. 10.1098/rsta.2006.1783 16766357

[B143] Pueyo E. Corrias A. Virág L. Jost N. Szél T. Varró A.. (2011). A multiscale investigation of repolarization variability and its role in cardiac arrhythmogenesis. Biophys. J. 101, 2892–2902. 10.1016/j.bpj.2011.09.060 22208187 PMC3244062

[B144] Ramanna H. Elvan A. Wittkampf F. H. de Bakker J. M. Hauer R. N. Robles de Medina E. O. (2001). Increased dispersion and shortened refractoriness caused by verapamil in chronic atrial fibrillation. J. Am. Coll. Cardiol. 37, 1403–1407. 10.1016/S0735-1097(01)01132-9 11300453

[B145] Ramirez R. J. Nattel S. Courtemanche M. (2000). Mathematical analysis of canine atrial action potentials: rate, regional factors, and electrical remodeling. Am. J. Physiol. Heart Circ. Physiol. 279, H1767–H1785. 10.1152/ajpheart.2000.279.4.H1767 11009464

[B146] Ravagli E. Bucchi A. Bartolucci C. Paina M. Baruscotti M. DiFrancesco D.. (2016). Cell-specific Dynamic Clamp analysis of the role of funny If current in cardiac pacemaking. Prog. Biophys. Mol. Biol. 120, 50–66. 10.1016/j.pbiomolbio.2015.12.004 26718599

[B147] Ravens U. Katircioglu-Öztürk D. Wettwer E. Christ T. Dobrev D. Voigt N.. (2015). Application of the RIMARC algorithm to a large data set of action potentials and clinical parameters for risk prediction of atrial fibrillation. Med. Biol. Eng. Comput. 53, 263–273. 10.1007/s11517-014-1232-0 25466224

[B148] Rees C. Yang J.-H. Santolini M. Lusis A. J. Weiss J. N. Karma A. (2018). Variability and compensation of cardiomycoyte ionic conductances at the population level. bioRxiv [Preprint]. 10.1101/283275

[B149] Rivolta I. Clancy C. E. Tateyama M. Liu H. Priori S. G. Kass R. S. (2002). A novel SCN5A mutation associated with long QT-3: altered inactivation kinetics and channel dysfunction. Physiol. Genomics 10, 191–197. 10.1152/physiolgenomics.00039.2002 12209021

[B150] Roberts B. N. Yang P. C. Behrens S. B. Moreno J. D. Clancy C. E. (2012). Computational approaches to understand cardiac electrophysiology and arrhythmias. Am. J. Physiol. Heart Circ. Physiol. 303, H766–H783. 10.1152/ajpheart.01081.2011 22886409 PMC3774200

[B151] Roden D. M. (2008). Repolarization reserve: a moving target. Circulation 118, 981–982. 10.1161/CIRCULATIONAHA.108.798918 18765386

[B152] Romero L. Carbonell B. Trenor B. Rodríguez B. Saiz J. Ferrero J. M. (2011). Systematic characterization of the ionic basis of rabbit cellular electrophysiology using two ventricular models. Prog. Biophys. Mol. Biol. 107, 60–73. 10.1016/j.pbiomolbio.2011.06.012 21749896

[B153] Romero L. Pueyo E. Fink M. Rodríguez B. (2009). Impact of ionic current variability on human ventricular cellular electrophysiology. Am. J. Physiol. Heart Circ. Physiol. 297, H1436–H1445. 10.1152/ajpheart.00263.2009 19648254

[B154] Rosati B. Dong M. Cheng L. Liou S. R. Yan Q. Park J. Y.. (2008). Evolution of ventricular myocyte electrophysiology. Physiol. Genomics 35, 262–272. 10.1152/physiolgenomics.00159.2007 18765860 PMC2585018

[B155] Rosati B. Mckinnon D. (2004). Regulation of ion channel expression. Circ. Res. 94, 874–883. 10.1161/01.RES.0000124921.81025.1F 15087427

[B156] Rose T. (2017). End of Average. London: Penguin Books.

[B157] Sadrieh A. Mann S. A. Subbiah R. N. Domanski L. Taylor J. A. Vandenberg J. I.. (2013). Quantifying the origins of population variability in cardiac electrical activity through sensitivity analysis of the electrocardiogram. J. Physiol. 591, 4207–4222. 10.1113/jphysiol.2013.251710 23551947 PMC3779112

[B158] Sale H. Wang J. O'Hara T. J. Tester D. J. Phartiyal P. He J. Q.. (2008). Physiological properties of hERG 1a/1b heteromeric currents and a hERG 1b-specific mutation associated with Long-QT syndrome. Circ. Res. 103, e81–95. 10.1161/CIRCRESAHA.108.185249 18776039 PMC2761010

[B159] Sampson K. J. Iyer V. Marks A. R. Kass R. S. (2010). A computational model of Purkinje fibre single cell electrophysiology: implications for the long QT syndrome. J. Physiol. 588, 2643–2655. 10.1113/jphysiol.2010.187328 20498233 PMC2916994

[B160] Sánchez C. Bueno-Orovio A. Wettwer E. Loose S. Simon J. Ravens U.. (2014). Inter-subject variability in human atrial action potential in sinus rhythm versus chronic atrial fibrillation. PLoS ONE 9:e105897. 10.1371/journal.pone.0105897 25157495 PMC4144914

[B161] Sarkar A. X. Christini D. J. Sobie E. A. (2012). Exploiting mathematical models to illuminate electrophysiological variability between individuals. J. Physiol. 590, 2555–2567. 10.1113/jphysiol.2011.223313 22495591 PMC3424714

[B162] Sarkar A. X. Sobie E. A. (2010). Regression analysis for constraining free parameters in electrophysiological models of cardiac cells. PLoS Comput. Biol. 6:e1000914. 10.1371/journal.pcbi.1000914 20824123 PMC2932676

[B163] Sarkar A. X. Sobie E. A. (2011). Quantification of repolarization reserve to understand interpatient variability in the response to proarrhythmic drugs: a computational analysis. Heart Rhythm 8, 1749–1755. 10.1016/j.hrthm.2011.05.023 21699863 PMC3202650

[B164] Sato S. Yamauchi S. Schuessler R. B. Boineau J. P. Matsunaga Y. Cox J. L. (1992). The effect of augmented atrial hypothermia on atrial refractory period, conduction, and atrial flutter/fibrillation in the canine heart. J. Thorac. Cardiovasc. Surg. 104, 297–306. 1495290

[B165] Schram G. Pourrier M. Melnyk P. Nattel S. (2002). Differential distribution of cardiac ion channel expression as a basis for regional specialization in electrical function. Circ. Res. 90, 939–950. 10.1161/01.RES.0000018627.89528.6F 12016259

[B166] Schulz D. J. Goaillard J. M. Marder E. (2006). Variable channel expression in identified single and electrically coupled neurons in different animals. Nat. Neurosci. 9, 356–362. 10.1038/nn1639 16444270

[B167] Schulz D. J. Goaillard J. M. Marder E. E. (2007). Quantitative expression profiling of identified neurons reveals cell-specific constraints on highly variable levels of gene expression. Proc. Natl. Acad. Sci. U.S.A. 104, 13187–13191. 10.1073/pnas.0705827104 17652510 PMC1933263

[B168] Sepulveda N. G. Roth B. J. Wikswo J. P. (1989). Current injection into a two-dimensional anisotropic bidomain. Biophys. J. 55, 987–999. 10.1016/S0006-3495(89)82897-8 2720084 PMC1330535

[B169] Sham J. S. Hatem S. N. Morad M. (1995). Species differences in the activity of the Na(+)-Ca2+ exchanger in mammalian cardiac myocytes. J. Physiol. 488 (Pt 3), 623–631. 10.1113/jphysiol.1995.sp020995 8576853 PMC1156729

[B170] Singh S. Zoble R. G. Yellen L. Brodsky M. A. Feld G. K. Berk M.. (2000). Efficacy and safety of oral dofetilide in converting to and maintaining sinus rhythm in patients with chronic atrial fibrillation or atrial flutter: the symptomatic atrial fibrillation investigative research on dofetilide (SAFIRE-D) study. Circulation 102, 2385–2390. 10.1161/01.CIR.102.19.2385 11067793

[B171] Skibsbye L. Jespersen T. Christ T. Maleckar M. M. Van den Brink J. Tavi P.. (2016). Refractoriness in human atria: time and voltage dependence of sodium channel availability. J. Mol. Cell. Cardiol. 101, 26–34. 10.1016/j.yjmcc.2016.10.009 27773652

[B172] Sobie E. A. (2009). Parameter sensitivity analysis in electrophysiological models using multivariable regression. Biophys. J. 96, 1264–1274. 10.1016/j.bpj.2008.10.056 19217846 PMC2717232

[B173] Soltis A. R. Saucerman J. J. (2010). Synergy between CaMKII Substrates and beta-Adrenergic Signaling in Regulation of Cardiac Myocyte Ca^2+^ Handling. Biophys. J. 99, 2038–2047. 10.1016/j.bpj.2010.08.016 20923637 PMC3042590

[B174] Soor N. Morgan R. Varela M. Aslanidi O. V. (2016). Towards patient-specific modelling of lesion formation during radiofrequency catheter ablation for atrial fibrillation. Conf. Proc. IEEE Eng. Med. Biol. Soc. 2016, 489–492. 10.1109/EMBC.2016.7590746 28261003 PMC5328409

[B175] Soylu M. Demir A. D. Ozdemir O. Soylu O. Topaloglu S. Kunt A.. (2003). Increased dispersion of refractoriness in patients with atrial fibrillation in the early postoperative period after coronary artery bypass grafting. J. Cardiovasc. Electrophysiol. 14, 28–31. 10.1046/j.1540-8167.2003.02218.x 12625606

[B176] Su Z. Li F. Spitzer K. W. Yao A. Ritter M. Barry W. H. (2003). Comparison of sarcoplasmic reticulum Ca^2+^-ATPase function in human, dog, rabbit, and mouse ventricular myocytes. J. Mol. Cell. Cardiol. 35, 761–767. 10.1016/S0022-2828(03)00119-6 12818566

[B177] Syed Z. Vigmond E. Nattel S. Leon L. J. (2005). Atrial cell action potential parameter fitting using genetic algorithms. Med. Biol. Eng. Comput. 43, 561–571. 10.1007/BF02351029 16411628

[B178] Taneja T. Mahnert B. W. Passman R. Goldberger J. Kadish A. (2001). Effects of sex and age on electrocardiographic and cardiac electrophysiological properties in adults. Pacing Clin. Electrophysiol. 24, 16–21. 10.1046/j.1460-9592.2001.00016.x 11227963

[B179] ten Tusscher K. H. Noble D. Noble P. J. Panfilov A. V. (2004). A model for human ventricular tissue. Am. J. Physiol. Heart Circ. Physiol. 286, H1573–H1589. 10.1152/ajpheart.00794.2003 14656705

[B180] ten Tusscher K. H. Panfilov A. V. (2006). Alternans and spiral breakup in a human ventricular tissue model. Am. J. Physiol. Heart Circ. Physiol. 291, H1088–H1100. 10.1152/ajpheart.00109.2006 16565318

[B181] Tobin A. E. Cruz-Bermúdez N. D. Marder E. Schulz D. J. (2009). Correlations in ion channel mRNA in rhythmically active neurons. PLoS ONE 4:e6742. 10.1371/journal.pone.0006742 19707591 PMC2727049

[B182] Tomaiuolo M. Bertram R. Leng G. Tabak J. (2012). Models of electrical activity: calibration and prediction testing on the same cell. Biophys. J. 103, 2021–2032. 10.1016/j.bpj.2012.09.034 23199930 PMC3491713

[B183] Trayanova N. A. (2014). Mathematical approaches to understanding and imaging atrial fibrillation significance for mechanisms and management. Circ. Res. 114, 1516–1531. 10.1161/CIRCRESAHA.114.302240 24763468 PMC4043630

[B184] Tsujimae K. Suzuki S. Murakami S. Kurachi Y. (2007). Frequency-dependent effects of various IKr blockers on cardiac action potential duration in a human atrial model. Am. J. Physiol. Heart Circ. Physiol. 293, H660–H669. 10.1152/ajpheart.01083.2006 17220183

[B185] Vagos M. R. Arevalo H. de Oliveira B. L. Sundnes J. Maleckar M. M. (2017). A computational framework for testing arrhythmia marker sensitivities to model parameters in functionally calibrated populations of atrial cells. Chaos 27, 093941. 10.1063/1.4999476 28964122

[B186] Vanier M. C. Bower J. M. (1999). A comparative survey of automated parameter-search methods for compartmental neural models. J. Comput. Neurosci. 7, 149–171. 10.1023/A:1008972005316 10515252

[B187] Vieira D.a,.G Adriano R. L. S. Vasconcelos J. A. Krahenbuhl L. (2004). Treating constraints as objectives in multiobjective optimization problems using niched pareto genetic algorithm. IEEE Trans. Magn. 40, 1188–1191. 10.1109/TMAG.2004.825006

[B188] Walmsley J. Rodriguez J. F. Mirams G. R. Burrage K. Efimov I. R. Rodriguez B. (2013). mRNA expression levels in failing human hearts predict cellular electrophysiological remodeling: a population-based simulation study. PLoS ONE 8:e56359. 10.1371/journal.pone.0056359 23437117 PMC3577832

[B189] Wang J. Liu L. Feng J. Nattel S. (1996). Regional and functional factors determining induction and maintenance of atrial fibrillation in dogs. Am. J. Physiol. 271, H148–H158. 10.1152/ajpheart.1996.271.1.H148 8760170

[B190] Wang W. Huang H. H. Kay M. Cavazos J. (2011). GPGPU accelerated cardiac arrhythmia simulations. Conf. Proc. IEEE Eng. Med. Biol. Soc. 2011, 724–727. 10.1109/IEMBS.2011.6090164 22254412 PMC3589987

[B191] Wang Z. Feng J. Nattel S. (1995). Idiopathic atrial fibrillation in dogs: electrophysiologic determinants and mechanisms of antiarrhythmic action of flecainide. J. Am. Coll. Cardiol. 26, 277–286. 10.1016/0735-1097(95)90845-F 7797763

[B192] Weaver C. M. Wearne S. L. (2008). Neuronal firing sensitivity to morphologic and active membrane parameters. PLoS Comput. Biol. 4:e11. 10.1371/journal.pcbi.0040011 18208320 PMC2211531

[B193] Weeke P. Mosley J. D. Hanna D. Delaney J. T. Shaffer C. Wells Q. S.. (2014). Exome sequencing implicates an increased burden of rare potassium channel variants in the risk of drug-induced long QT interval syndrome. J. Am. Coll. Cardiol. 63, 1430–1437. 10.1016/j.jacc.2014.01.031 24561134 PMC4018823

[B194] White J. W. Rassweiler A. Samhouri J. F. Stier A. C. White C. (2014). Ecologists should not use statistical significance tests to interpret simulation model results. Oikos 123, 385–388. 10.1111/j.1600-0706.2013.01073.x

[B195] Xiao L. Xiao J. Luo X. Lin H. Wang Z. Nattel S. (2008). Feedback remodeling of cardiac potassium current expression: a novel potential mechanism for control of repolarization reserve. Circulation 118, 983–992. 10.1161/CIRCULATIONAHA.107.758672 18711016

[B196] Yan G. X. Shimizu W. Antzelevitch C. (1998). Characteristics and distribution of M cells in arterially perfused canine left ventricular wedge preparations. Circulation 98, 1921–1927. 10.1161/01.CIR.98.18.1921 9799214

[B197] Yang P. C. Clancy C. E. (2012). *In silico* prediction of sex-based differences in human susceptibility to cardiac ventricular tachyarrhythmias. Front. Physiol. 3:360. 10.3389/fphys.2012.00360 23049511 PMC3442371

[B198] Yang P. C. El-Bizri N. Romero L. Giles W. R. Rajamani S. Belardinelli L.. (2016). A computational model predicts adjunctive pharmacotherapy for cardiac safety via selective inhibition of the late cardiac Na current. J. Mol. Cell. Cardiol. 99, 151–161. 10.1016/j.yjmcc.2016.08.011 27545042 PMC5453509

[B199] Yang P. C. Kurokawa J. Furukawa T. Clancy C. E. (2010). Acute effects of sex steroid hormones on susceptibility to cardiac arrhythmias: a simulation study. PLoS Comput. Biol. 6:e1000658. 10.1371/journal.pcbi.1000658 20126530 PMC2813260

[B200] Yang P. C. Perissinotti L. L. López-Redondo F. Wang Y. Demarco K. R. Jeng M. T.. (2017). A multiscale computational modelling approach predicts mechanisms of female sex risk in the setting of arousal-induced arrhythmias. J. Physiol. 595, 4695–4723. 10.1113/JP273142 28516454 PMC5509858

[B201] Zaniboni M. (2011). 3D current-voltage-time surfaces unveil critical repolarization differences underlying similar cardiac action potentials: a model study. Math. Biosci. 233, 98–110. 10.1016/j.mbs.2011.06.008 21781977

[B202] Zhang Z. S. Tranquillo J. Neplioueva V. Bursac N. Grant A. O. (2007). Sodium channel kinetic changes that produce Brugada syndrome or progressive cardiac conduction system disease. Am. J. Physiol. Heart Circ. Physiol. 292, H399–H407. 10.1152/ajpheart.01025.2005 16877553

[B203] Zhao J. Hansen B. J. Wang Y. Csepe T. A. Sul L. V. Tang A.. (2017). Three-dimensional integrated functional, structural, and computational mapping to define the structural “fingerprints” of heart-specific atrial fibrillation drivers in human heart *ex vivo*. J. Am. Heart. Assoc. 6:e005922. 10.1161/JAHA.117.005922 28862969 PMC5586436

[B204] Zhou J. Kodirov S. Murata M. Buckett P. D. Nerbonne J. M. Koren G. (2003). Regional upregulation of Kv2.1-encoded current, IK,slow2, in Kv1DN mice is abolished by crossbreeding with Kv2DN mice. Am. J. Physiol. Heart Circ. Physiol. 284, H491–H500. 10.1152/ajpheart.00576.2002 12529256

[B205] Zhou Q. Zygmunt A. C. Cordeiro J. M. Siso-Nadal F. Miller R. E. Buzzard G. T.. (2009). Identification of Ikr kinetics and drug binding in native myocytes. Ann. Biomed. Eng. 37, 1294–1309. 10.1007/s10439-009-9690-5 19353268 PMC2690829

[B206] Zhou X. Bueno-Orovio A. Orini M. Hanson B. Hayward M. Taggart P.. (2016). *In vivo* and *in silico* investigation into mechanisms of frequency dependence of repolarization alternans in human ventricular cardiomyocytes. Circ. Res. 118, 266–278. 10.1161/CIRCRESAHA.115.307836 26602864 PMC4719495

[B207] Zhu Z. I. Clancy C. E. (2007). Genetic mutations and arrhythmia: simulation from DNA to electrocardiogram. J. Electrocardiol. 40, S47–50. 10.1016/j.jelectrocard.2007.05.033 17993328

